# Revolutionizing healthcare with nanosensor technology

**DOI:** 10.1039/d5ra04412j

**Published:** 2025-10-16

**Authors:** Nakshatra Bahadur Singh, Asima Monjur, Bhuvnesh Kumar, Shivani Varshney, Md. Abu Bin Hasan Susan

**Affiliations:** a Research Development Cell, Sharda University Greater Noida India n.b.singh@sharda.ac.in; b Department of Chemistry, University of Dhaka Dhaka-1000 Bangladesh susan@du.ac.bd; c Department of Science and Humanities, Military Institute of Science and Technology Mirpur Cantonment Dhaka-1216 Bangladesh; d Micron Technology, Inc. Boise Idaho 83716 USA; e Dhaka University Nanotechnology Centre, University of Dhaka Dhaka-1000 Bangladesh

## Abstract

Modern healthcare is constantly evolving due to the inclusion of many smart technologies. Nanotechnology, since its inception, has tremendous influence on this transformation. With an emphasis on healthcare applications, the current review explores recent advancements in nanomaterial-based biosensors. The tunability of nanomaterials provides control over the chemical, mechanical, thermal, and electrical properties, thus presents nanotechnology as a more promising solution to develop smart sensing, monitoring, and diagnostics. The synergistic effects and regulated interaction with a variety of bioanalytes spurred the advancements of nano empowered devices with high selectivity and sensitivity. With a focus on common nanomaterials *e.g.*, metal and metal oxides, graphene and polymer-based nanomaterials, the review comprehensively discusses fundamentals of nano enabled biosensors and their classification based on methods of detection. Insight has been provided exploring the potential of various types of nanomaterials harnessed in the development of pioneering sensor designs in recent times. Along with addressing the current limitations, future prospects are discussed to redefine the landscape of nanobiosensors.

## Abstract 2

The review highlights the latest developments in nanotechnology enabled sensors in healthcare applications with a focus on the potential of nanomaterials harnessed in their fabrication.

## Graphical abstract

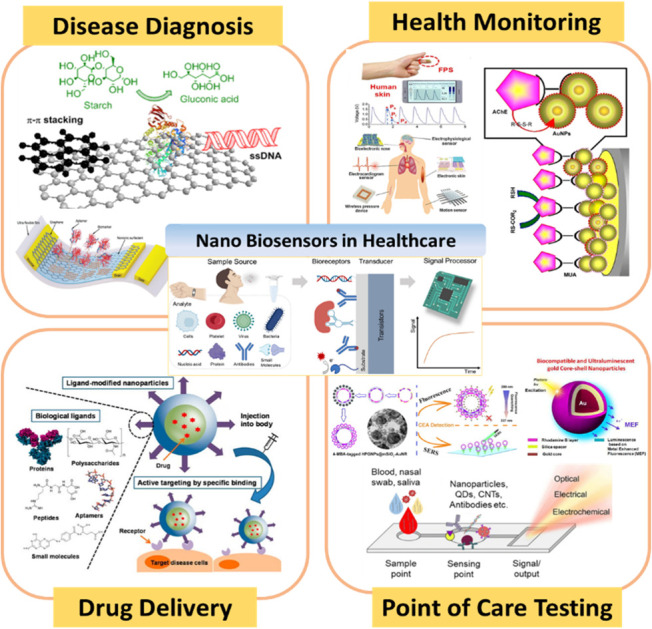

## 1. Introduction

Intelligent therapies, advanced analytical tools, and smart diagnostic systems are the prerequisites to reach smart health care management benchmarks. It is undeniable that success of treatments of infectious disease and survival depend largely on the detection of diseases at an early stage. Point of care devices (PoCs) and biosensing techniques for biomolecular quantification in a number of diseases have been crucial in recent decades in improving the standard of clinical urgency in the medical field. Despite the availability of successful and inexpensive analytical methods, sophisticated instrumentation makes the real time detection far more difficult. Sometimes the minuscule presence of biomarkers in physiological fluids is another barrier. Nanomaterials – the heart of the nanosensor with their promising characteristics contribute significantly in a broad spectrum of sensing applications particularly in health sector. Promising detection and sensing, user-friendliness and reliable nature is accountable for their unprecedented popularity. The large surface-to-volume ratio of nanoscale materials produces distinctive physicochemical properties enabling them to have specific interaction capabilities with biomolecules, a strong foundation for the development of next-generation diagnostic devices. Any devices for *in situ* monitoring need not only to be simpler in structure but should also provide high sensitivity, and selectivity during measurement.

**Fig. 1:**
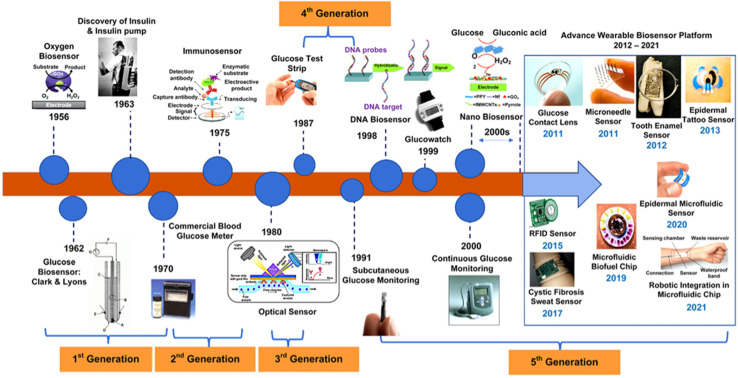
Different milestones in the field of biosensors.^22^

Health monitoring involves sensors that usually operates through a biological reaction mediated by enzymes, organelles, cells, tissues or immunosystems and detect any chemical compounds by optical, electrical or thermal signals. These bioanalytical devices involve a molecular recognition entity along with a physicochemical transducer. In this context, nanosensors are recent addition with their miniature dimensions and high sensitivity, selectivity, and responsiveness. The exceptional performance is attributed to nanoscale phenomena of atoms, that act as the building block of the sensors. Three crucial components for a biosensor are – a bioreceptor to recognize the interaction with analyte and change its properties, a transducer to translate the interaction, and an amplifier to amplify the signal and provide data in a readable form. In general, enzymes,^1–3^ antibodies,^4,5^ DNA/RNA,^6–8^ and aptamers^9–12^ are commonly used as bioreceptors, whereas the biochemical interaction is detected by means of optical,^13,14^ electrochemical,^15–17^ acoustic,^18,19^ and thermal^20,21^ signals. Recent developments in nanomaterial (NM) synthesis are greatly spurred from their potential to use as the receptor or transducing element of such biosensors. A great deal of effort has been put forward in order to improve their inherent properties, create stable surface chemistries, and integrating them into sensing devices. Evolution of different types of biosensors for different applications are represented in Fig. 1.^22^

Evolution of bionanosensors can be distinguished into five different generations, each propelled by advancements in signal transduction, bioreceptor technology, and device integration. The simplest concept of sensing can be found among first generation biosensors which is the direct interaction of analytes and products. The concept of biosensing was first established with the fabrication of oxygen electrode by Leland C. Clark Jr in 1956 to measure oxygen concentration in blood.^23^ In 1962, more functionality in transduction was achieved by direct electrochemical detection of analytes; as a part of this attempt electrode was conjugated with enzyme using a dialysis membrane. Modification of Clark's work came in the form of glucose oxidase immobilized oxygen sensor.^24^ However, these electrodes had poor stability and were highly susceptible to interference by endogenous electroactive species. In 1963, first subcutaneous insulin pump by Dean Kamen became commercialized.^25^ In contrast to first-generation biosensors, second-generation biosensors included redox mediators the role of which is to shuttle electrons directly between the active site of enzyme and electrode. Followed by this, the first portable blood glucose meter ‘Ames Reflectance Meter’ was commercialized.^26–28^ This was used in combination with the Dextrostix, apparently required small volume of blood like typical POC diagnostics. Potentiometric enzyme electrodes were subsequently introduced by Guilbault and Montalvo in 1969 for the targeted detection of urea.^29^ The exploitation of various transduction mechanism was initiated even though, their commercialization was challenging.^30,31^ This can be exemplified by “thermistor” developed by the group of Klaus Mosbach.^32^ Third generation biosensors all include bioreceptor, however, the sensitivity has been improved by the direct interface between bioreceptor and electrodes. In addition, low cost and feasibility of repeated measurements are achieved in these biosensors. For example, addition of auxiliary enzymes and co-reactants brought out the pH electrodes and ion-selective field-effect transistors with improved analytical efficiency compared to their predecessors.^33,34^ In 1974, enzyme thermistors and thermal enzyme probes were introduced which involves heat transduction.^35,36^ However, successful monitoring of biomolecular interactions in immunodiagnostics delve from the use of optical biosensors, which operate on the principles of fluorescence, absorbance, or refractive index. Meanwhile, a wide variety of bioreceptor–transducer combinations were introduced exploiting enzymes, cell receptors, nucleic acids, antibodies, in combination with electrochemical, piezoelectric, magnetic, thermometric, and micromechanical transducers.^37–39^ Instead of natural cofactors or artificial mediators for signal transduction, third-generation sensors relied on direct electron transfer between the biorecognition element and the electrode. Nanosensors utilize molecular phenomena to detect real time minor changes. This advance was made possible largely due to the unique physicochemical properties of nanomaterials (NMs). The properties of materials largely differ with particle size, dimensions, chemical composition, and morphology in nanoscale, thus make it easy to use them in sensors for the detection of particular analyte. A wide range of NMs have been used to develop nanoscale biosensors: from organic to polymeric nanostructures such as micelles, dendrimers, carbon nanotubes, solid lipid nanoparticles (NPs), graphene, liposomes, *etc.* Catalytic properties, electron transition, and high surface to volume ratio enhance the performance, selectivity, and responsiveness of nanosensors. As a result, a significant shift is observed from single-use test devices toward real-time and continuous monitoring system. Especially the nanobiosensors for continuous glucose monitoring platforms (2000s) enabled real-time and label-free detection. With that essence, fifth generation of sensors focuses on flexible and wearable technologies, such as robotic-integrated chips, microneedle sensors, epidermal tattoo sensors, and microfluidic platforms. These technologies integrate nanosensors with digital health, Internet of Things (IoT), and artificial intelligence ecosystems to provide personalized and non-invasive healthcare solutions.

While most current reviews emphasize recent advances in sensor technologies for healthcare, this work presents a holistic approach by highlighting the pivotal role of NMs in the evolution of biosensors and their transformative impact on biomedicine. Along with the comprehensive discussion on the current landscape of sensing technologies, important application of biosensors has also been discussed for readers with an aim of having nuanced understanding of potential of NMs. By delving into the intricacies of three key aspects – safety and environmental considerations, clinical applications and the global regulatory landscape, prospect and key challenges in fabrication and implementation of such sensors are also discussed. Considering the promising ability of NM enabled biosensors in medicine and diagnostics, it is foreseeable that they will provide an efficient and reliable platform for early disease diagnostics in near future.

## 2. Nanomaterials used in nanosensors

With increasing costs and effective patient-centric care, nanosensors have become effective tool and a cutting-edge solution that could reshape diagnostics, therapeutics, and preventive medicine. As a large number of atoms are located near the surface of a NM, a remarkable difference is observed in their physicochemical properties. There is also quantum confinement of delocalised electrons, which produces discontinuity in their behaviour. They tend to do so by acting as immobilization platforms, enhance refractive index changes, magnify mass changes, catalyze reactions between substrates and chemiluminescent, and accelerate electron transfer.

**Fig. 2:**
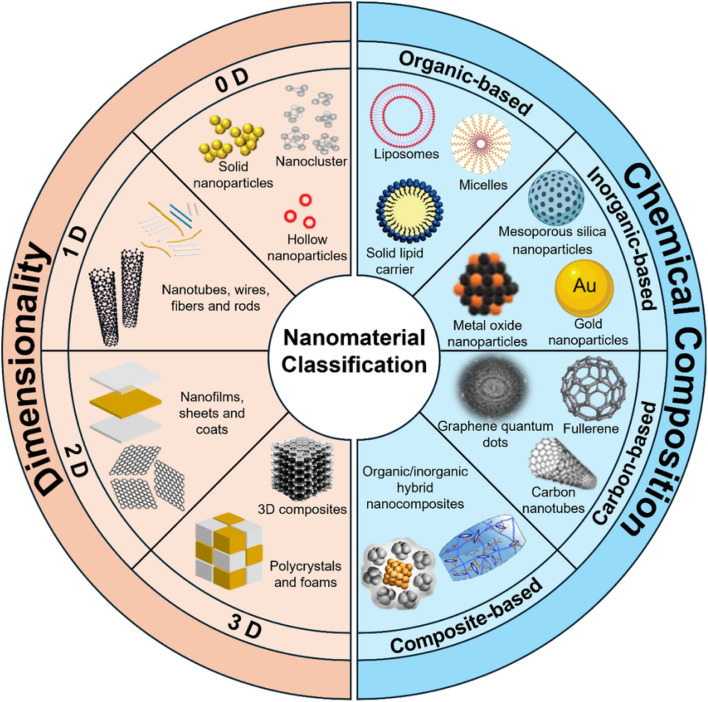
Classification of nanomaterials used in biosensors for healthcare application.^40^

There can be a number of basis to classify NMs used in biosensing such as their spatial confinement, porosity, origin, phase and dispersion. This review emphasizes on the biosensors based on the abundant NMs, as showed in Fig. 2.^40^ They are broadly classified as – (a) inorganic: metal and metal oxide derived NPs, (b) organic: micelles, dendrimers and liposomes, (c) carbon-based: graphene and graphene oxide, single and multi-walled carbon nanotubes (CNTs). Biosensors are later classified in terms of the dimension of NMs used. However, the distinct advantages that NMs from various sources offer determine how they are used in biosensors for the detection of bioanalytes.

Organic NM encompasses micelles, dendrimers, liposomes, metal organic frameworks that are formed from covalent and non-covalent assemblies of organic molecules. They are highly preferable for *in vivo* testing for biocompatible nature. Nevertheless, their low mechanical strength and stability are circumvented by inorganic NPs. Inorganic NMs typically include Au, Ag, Al, Cu, Fe, Zn, and Pb NPs and their oxides. Furthermore, because of their extraordinary surface area, tunable chemistry, layered double hydroxides (LDHs), metal carbides and nitrides (MXenes), and 2D transition metal borides (MBenes) are becoming more and more promising and potential for drug delivery, biosensing, and theranostics. Carbon allotropes provide an unparalleled combination of mechanical, electrical, and optical characteristics that result in miniature sensors with excellent performance and low power needs. Occasionally, the attachment of biomolecules is a prerequisite to enhance the molecular recognition, which opens up the avenue of surface engineering of these NMs by covalent or non-covalent modifications in order for the immobilization of a range of biomolecules namely aptamers, enzymes, viruses, antibodies.^41^ Carbon nanotubes emerge as scaffold materials for immobilisation of biomolecules, thus ameliorate the molecular recognition. Chemical reactivity of carbon nanotube increases 1000 times with an increase in surface area per unit.^42^ Graphene and their derivatives with their packed hexagonal structure, act as an anchor site for physisorption of analytes. Table 1 presents a summary of the key properties of aforementioned NMs used in biosensors.

**Table 1:** Different types of nanomaterials used in biosensors in healthcare application

Category of nanomaterials	Nanomaterials	Example	Key properties
Inorganic NMs	Metal NPs	Au, Ag, Pt, and Pd NPs	-High electrical conductivity
			-Strong surface plasmon resonance
			-High catalytic activity
			-Easy surface functionalization
			-Biocompatible
	Metal oxide NPs	ZnO, TiO_2_, Fe_3_O_4_, CuO, SnO_2_	-Semiconducting properties
			-Magnetic properties
			-Photocatalytic activity
			-Cost-effectiveness and chemically stability
	MXenes (2D transition metal carbides/nitrides)	Ti_3_C_2_T_*x*_, Nb_2_C, Mo_2_C	-High conductivity
			-High hydrophilicity and biocompatibility
			-Strong electrochemical activity
			-Mechanical flexibility
Carbon-based NMs	Carbon nanotubes	Single-walled and multi-walled	-High electrical conductivity
			-Large surface area
			-High mechanical strength
			-Ease of surface functionalization
	Graphene and graphene oxide (GO, rGO)	Monolayer graphene, GO, reduced GO	-Ultra-high electrical conductivity
			-High surface-to-volume ratio
			-Tunable surface chemistry
			-Biocompatibility and mechanical flexibility
			-Fluorescence quenching
	Fullerenes (C_60_ and derivatives)	C_60_, functionalized fullerenes	-Unique spherical structure
			-High electron-accepting ability
			-Good redox properties
			-High stability
			-Strong photoluminescence
	Quantum dots (QDs) and carbon dots	Graphene QDs, carbon dots	-Size-tunable photoluminescence
			-High dispersibility and biocompatibility
			-High surface area
			-Low toxicity
			-Fluorescence resonance energy transfer capability
Polymer-based NMs	Conducting polymers	Polyaniline, polypyrrole, poly(3,4-ethylenedioxythiophene)	-High electrical conductivity
			-Film-forming ability
			-Stable in physiological conditions
			-Easy to functionalize for biomolecule attachment
	Biodegradable polymers	PLGA, chitosan, polycaprolactone	-Biocompatibility
			-Presence of surface functional groups
			-Able to encapsulate drugs
			-Non-toxicity, safe for *in vivo* use
	Molecularly imprinted polymers	Acrylamide, methacrylic acid, polyurethane-derivatives	-Mimicking natural receptors
			-High selectivity and stability
			Stability in harsh environments
			-Cost-effectiveness

Recently in healthcare especially for drug delivery, interest in polymeric NMs has escalated. Exceptional biocompatibility, inertness, thermal stability, and flexibility in design and fabrication are some of the reasons behind it. Polymeric cross-linked NPs are able to conjugate to any biomolecule in a selective manner, acting as a promising biomimetic material for antibody receptor. 3D polymeric networks and the hyperbranched structures of dendrimers allow tuning of exterior surface in response to change in external environment such as in pH, magnetic or electric field. Table 2 represent a short overview of biosensors developed in recent times harnessing the power of NMs.^22^

**Table 2:** Nanomaterials used in biosensor development over the last two decades^22^

Transduction mechanism	Nanomaterial	Analyte	Detection limit	Linear range
Electrochemical	Au NPs	Uranyl	0.3 μg L^−1^	2.4–480 μg L^−1^
	Au/CdS QDs/TNTs	Cholesterol	0.012 μM	0.024–1.2 mM
		H_2_O_2_	0.06 μM	18.73–355.87 μm
	Au NPs	*E. coli*	15 CFU mL^−1^	10–106 CFU mL^−1^
	Au/rGO	miENA-122	1.73 pm	10 μm–10 pm
	Au NPs/TiO_2_	H_2_O_2_	5 μm	65–1600 μm
	Ag/Pd NPs	Ractopamine	1.52 pg mL^−1^	0.01–100 ng mL^−1^
		Clenbuterol	1.44 pg mL^−1^	0.01–100 ng mL^−1^
		Salbutamol	1.38 pg mL^−1^	0.01–100 ng mL^−1^
	Ag@CQDs-rGO	Dopamine	0.59 nm	0.1–300 μm
	Ag NP-MWNT	Glucose	0.01 mM	0.025–1.0 mM
	Pt NPs/RGO–CS–Fc	H_2_O_2_	20 nm	2.0 × 10^−8^–−3.0 × 10^−8^ M
	Pt@CeO_2_ NM	Dopamine	0.71 nM	2–180 nM
	Pd/Co-NCNT	Hydrazine	0.007 μm	0.05–406.045 μm
	Pd/CNF/[M3OA]^+^[NTF2]^−^	H_2_	0.33 nM	1.00–35.0 nM
	Cu/rGO-BP	Glucose	11 μm	0.1–2 mM
	NiO@Au	Lactic acid	11.6 μM	100.0 μM–0.5 M
	Co_3_O_4_ NCs	Glutamate	10 μM	10–600 μM
	Fe_2_O_3_/NiO/Mn_2_O_3_ NPs	Folic acid	96.89 ± 4.85 pM	0.1 nM–0.01 mM
	MoO_3_@RGO	Breast cancer	0.001 ng mL^−1^	0.001–500 ng mL^−1^
	G/Au NR/PT	HPV DNA	4.03 × 10^−14^ M	1.0 × 10^−13^–1.0 × 10^−10^ M
	Graphene QDs	Cu^2+^	1.34 nM	0.015–8.775 μM
	LAC-CNTs-SPCE	*Para*-cresol	0.05 ppm	0.2–25 ppm
	GQDs-MWCNTs	Dopamine	0.87 nM	0.005–100.0 μM
Electrochemiluminescence	Ag NPs	Mucin 1	0.37 fg mL^−1^	1.135 fg mL^−1^–0.1135 ng mL^−1^
	Au NPs@PDA@CuInZnSQDs	P53 gene	0.03 nM	0.1–15 nM
Photoelectricalchemical	Co_3_O_4_–Au	miRNA-141	0.2 pM	1 pM–50 nM
	TiO_2_ NTs	Asulam	4.1 pg mL^−1^	0.02–2.0 ng mL^−1^
	Co_3_O_4_-CNT/TiO_2_	Glucose	0.16 μM	0–4 mM
Amperometry	Pt–Fe_3_O_4_@C	Sarcosine	0.43 μm	0.5–60 μm
	Cu_2_O@CeO_2_–Au	PSA	0.0001–100.0 ng mL^−1^	0.03 pg mL^−1^
	NiO/PANINS	Glucose	0.06 μM	1–3000 μM
	Graphene-Au NRs	NADH ethanol	6 μM	20–160 μM
			1.5 μM	5–377 μM
	CNT/Au NPs	Choline	15 μM	0.05–0.8 mM
Voltammetry	Pt NFs/PAni	Urea	10 μm	20 mM
	Cu NPs/Rutin/MWCNTs/IL/Chit/GCE	H_2_O_2_	0.11 μm	0.35–2500 μM
	Cr doped SnO_2_ NPs	Riboflavin	107 nM	0.2 × 10^−6^–1.0 × 10^−4^ M
Impedimetry	MnO–Mn_3_O_4_@rGO	H_2_O_2_	0.1 μM	0.004–17 mM
	MnO_2_ NFs	Salmonella	19 CFU mL^−1^	3.0 × 10^1^–3.0 × 10^6^
	TiO_2_/APTES	Glucose	24 μmol	50–1000 μmol
	Au NBPs	Aflatoxin B1 (AFB1)	0.1 nM	0.1–500 nM
Fluorescence	Au NPs	Pb^2+^	16.7 nm	50 nm–4 μm
	Graphene QDs	Lung cancer^+^	0.09 pg mL^−1^	0.1 pg ml^−1^–1000 ng mL^−1^
	CdTe/CdS//ZnS core/shell/shell QDs	l-Ascorbic acid	1.8 × 10^−9^ M	8.0 × 10^−9^–1.0 × 10^−7^ M
	ZnO-rGO	Dopamine	8.75 ± 0.64 pM	0.1–1500 pM
	NSETamptamer@Fe_3_O_4_@GOD and MoS_2_	Tumor cell (EpCAM)	1.19 nM	2–64 nM
FET	Ni/Cu MOF	Glucose	0.51 μM	1 μM–20 mM
	ZnO NRs	Phosphate	0.5 mM	0.1 μM–7.0 mM
	CaM/SiNW	Protein	7 nM	10^−8^–10^−6^ M
	FETs			
	Si NWs	Dengue virus	2.0 fM	1 μM–10 fM
	ZnO NRs	Phosphate	0.5 mM	0.1 μM–7.0 mM
SAW	Au NP-MoS_2_-rGO	Carcinoembryonic antigen (CEA)	0.084 ng mL^−1^	36.58 ng mL^−1^
Colorimetric	Ag NPs	H_2_O_2_	0.032 μm	0.05–7.5 μm
		Glucose	0.29 μm	1.5–3.0 μm
		Fe^2+^	0.54 μm	1–90 μm
	Pt NPs	Adrenaline	2.93 × 10^−4^ M	9.99 × 10^−1^–2.13 × 10^−4^ mol L^−1^
SPR	SAM/NH_2_rGO/PAMAM	DENV 2E	0.08 pM	0.08–0.5 pM

## 3. Classification of nanosensors

**Fig. 3:**
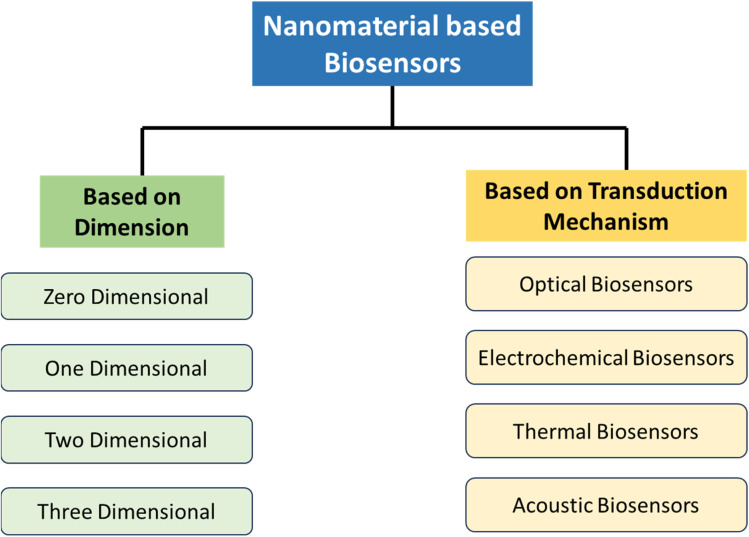
Classification of nanomaterial-based biosensors based on dimension and mechanism of signal transduction.

NMs can be classified based on a number of criteria such as their detection system, transducer technology, and nature of bio-receptors. Insights into the wide variety of biosensing techniques are provided by the classification in Fig. 3, which helps to find out the suitable biosensor for their particular application. As the emphasis here has been placed on the application of nanotechnology in healthcare, the classification of the biosensors has been shown based on the dimension of the NMs and their transduction and detection mechanism.

Various types of nanosensors have been discussed as follows.

### 3.1 Based on dimension of nanomaterials

Nanobiosensor devices can be distinguished into four groups based on the dimensions of the NMs: zero-dimensional (QDs), one-dimensional (thin films), two-dimensional (carbon nanotubes and nanorods), and three-dimensional (gold, and liposomes).

#### 3.1.1 Zero-dimensional nanostructures

Zero-dimensional nanostructures present a vast surface area. This large surface provides plenty of room for immobilizing biomolecules, facilitating effective interaction between sensing elements and target analytes. Their extensive applications are found in ultra-sensitive biosensing applications.

#### 3.1.2 One-dimensional NMs

One-dimensional nanostructures ensure efficient electron transport pathways which is essential for enhanced signal transduction. They are ideal choices for designing robust sensing infrastructures because of the quick response and accuracy.

#### 3.1.3 Two-dimensional NMs

Two-dimensional nanostructures are characterized by specific planes for immobilization processes. They are highly valuable for multi-analyte biosensing at the same time. The versatility in structures not only enhances targeting ability of the sensor but also help distinguish analytes in complex samples.

#### 3.1.4 Three-dimensional NMs

Three-dimensional nanostructures provide additional advantages for immobilization with the help of their outer and inner surfaces. As a result, more biomolecules can be attached resulting in the improved sensitivity and accuracy of the biosensor.

### 3.2 Based on transduction mechanism

#### 3.2.1 Optical biosensor

The minuscule intrinsic changes in optical properties allow real-time and non-invasive monitoring of interactions. Optical biosensors can extensively be classified based on the interaction mechanisms between the recognition element and targets. These are affinity and metabolism sensors. Affinity sensors where operate through binding interactions between a target and a receptor such as aptamer-based sensing, antigen–antibody binding, complementary DNA hybridization, metabolic sensors use molecular recognition through enzymatic reactions. Thus, in addition with the detection, quantitative analysis is also possible. Morever, there are label free and label based optical sensors. In label-free optical biosensors interactions among biomolecules are traced directly, without attaching any fluorescent or chemical tags to the analyte. Contrary to that, label-based optical biosensors allow uses of external tags that produce a strong and measurable signal when bound to the target. Optical biosensor operates based on a number of principles in label-free and label-based detection, *e.g.*, chemiluminescence,^43,44^ fluorescence,^45,46^ and scattering.^47,48^ When it comes to nanoscale phenomena, localized surface plasmon resonance (LSPR) is one of the widely used techniques that involves collective oscillation of conduction electrons induced by incident light.^49,50^ The LSPR frequency of the metal NPs varies according to the local dielectric environment. The detection of analytes can be recognized from the shift in LSPR wavelengths or the change in the peak intensity. Binding of analytes on the surface of semiconductor NPs can also be understood from the shift in refractive index as well as from fluorescence light intensity. Fluorescence labelled biomolecules in these sensors involves fluorescent quenching, fluorescent enhancement and Förster resonance energy transfer (FRET).^51,52^ Contrary to it, surface enhanced Raman scattering (SERS) involves vibrational bonding information of scattered photons. Metal NPs or other plasmonic nanostructured surfaces show enhanced Raman scattering signals of analytes when they get adsorbed on the surfaces. Surface plasmon resonance (SPR) based biosensors use surface plasmon waves to measure the shift in refractive index brought on by molecule interaction at a metal surface.

#### 3.2.2 Electrochemical biosensor

This type of sensors is now abundant due to their simplicity, replicability, and minimal expense. The sensor monitors biochemical reaction that involves redox mechanism. When a redox biorecognition event occurs, such as the binding of an enzyme, antibody, aptamer, or DNA strand to its target, the interfacial properties of the electrode surface change, leading to measurable variations in electrical current, voltage, or impedance. If measured as a signal, such a variation of electrical properties can be correlated with the concentration of analyte. The change of current is also manifested for any materials catalyzing the reaction between receptor and analyte. In some cases, these materials can bind charged analytes leveraging the ion-gating effects. The differences in conductance between two metal electrodes is measured as a result of a biological variable in conductometric techniques. In potentiometric biosensors, the potential of an active electrode is compared to a reference electrode to detect changes as a result of any reaction. Voltammetry comprises of electro-analytical methods that gather information about the analyte by varying potential to measure the resulting current. Cyclic voltammetry, differential pulse voltammetry, and linear sweep voltammetry fall under this category. On the contrary, the impedimetric technique measures the frequency-dependent resistance of charge transfer at the electrode–electrolyte interface. Charge transfer resistance is particularly important in a sense that it allows quantitative detection of analytes in real samples.

#### 3.2.3 Thermal and acoustic biosensors

By measuring the heat change generated by a biological reaction between the analyte and the proper enzyme, calorimetric biosensors are able to identify analytes. Thermal biosensors are usually immobilized thermal reactors with different thermal transducers like thermopile and thermistors. While thermopiles measure the temperature gradient between two regions, thermistors measure temperature from the variations in electric resistance. Magnetic biosensors are developed utilizing magnetic NPs as scaffolds for isolation, purification aside sensing. Acoustic wave sensors, on the other hand, monitor any slight changes in the physical characteristics of acoustic waves produced and detect them using a variety of techniques, including magnetostrictive, piezoelectric, optical, and thermal. The adsorption of analytes on the anisotropic crystal sufficiently changes its piezoelectric properties, thus providing a means to monitor local environment from the variation in its fundamental frequency of oscillation. In both of the sensors, the role of various NMs is to increase the sensitivity wither by increasing heat conductivity or the sensitivity to detect minuscule changes in heat or acoustic waves. Antibody modified sol particles while bind on the electrode surface, the corresponding change in the vibrational frequency serves as a basis of detection. Field-effect transistors (FETs) regulate the flow of current through a semiconductor in presence of electric field. As a result, interaction between the analyte and FET surface can be interpreted from the change in the surface potential.

## 4. Fabrication of nanosensors

**Fig. 4:**
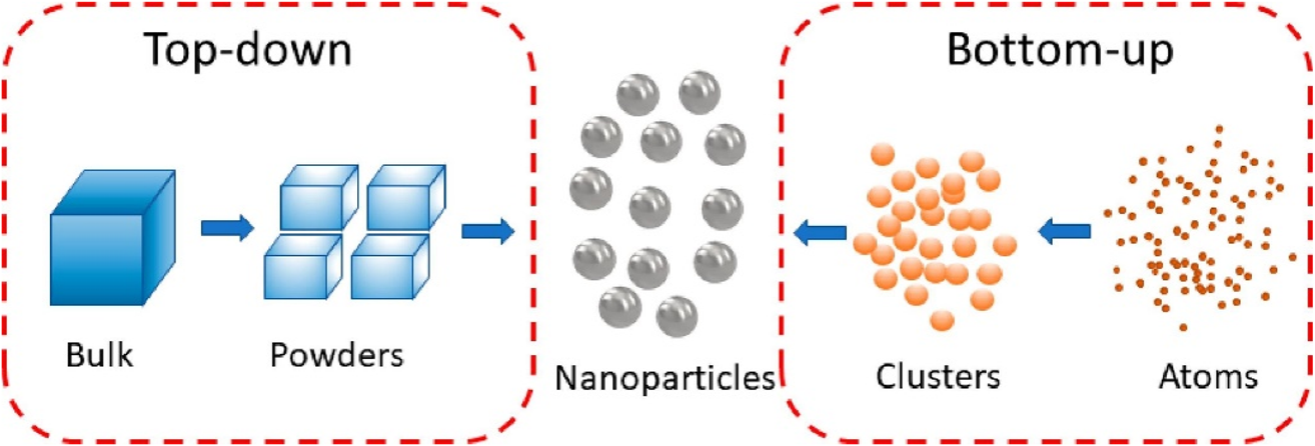
Bottom-up and top-down methods for synthesizing NMs.^53^

Nanofabrication techniques are used for the fabrication of nanosensors. This allows precise control over size, shape, and composition of the NMs. Common fabrication techniques are top-down (*e.g.*, ion beam milling, electron beam lithography) and bottom-up approaches (*e.g.*, deposition, self-assembly, *etc.*) (Fig. 4).^53^ These techniques produce nanosensors with high precision and reproducibility.

Because of the higher surface-to-volume ratio, more biorecognition elements can be attached to sensor surfaces. These molecules are mounted on the detecting surface in a way so that stable interaction with the analyte becomes possible. The selectivity and sensitivity of target analyte is enhanced by functionalization of nanosensors. Surfaces of nanosensors are changed by functionalization and can interact selectively with the analyte (DNA sequences, enzymes, antibodies, or molecularly imprinted polymers). Molecular attachment can be both-covalent or noncovalent in nature. For example, the binding of proteins on the surface of NMs is only possible by hydrophobic, van der Waals, electrostatic, and hydrogen bonding interactions.^54^ Thus, any changes in ionic strength, temperature of the local environment as well as the surface of NMs must have significant influence. However, after fabrication, rigorous testing of the nanosensors are made to evaluate their sensitivity, selectivity, response time, and performance. Once the nanosensors have been developed and optimized, they can be integrated into larger systems or devices for practical applications.

## 5. Different material systems for biosensing

### 5.1 Metal nanocomposite biosensors: bridging nanotechnology and biomedicine

The immense interest on metal and metal oxide NPs in recent years stems from their unique combination of physicochemical, electrical, optical, and catalytic properties. Au, Ag, Pt, and Pd NPs possess excellent electrical conductivity, high surface-to-volume ratio, and LSPR originated from large surface-to-volume ratio and highly crystalline nature. In parallel, metal oxide NPs *e.g.*, ZnO, TiO_2_, Fe_3_O_4_, CeO_2_, CuO exhibit semiconducting behaviour, high isoelectric points, a set of properties essential for biomolecule immobilization, electron transfer, and catalytic activity in sensing platforms. Of particular interest is their biocompatible nature, high robustness, and interaction with variety of organic molecules that made them effective as electrocatalysts. Metal oxide NMs can be attributed as materials to enhance performance in electrochemical biosensors. Oxides of nickel, copper, iron, cobalt NPs are used in electrochemical sensor for on-site detection of glucose, 4-acetaminophen, uric acid, and cholesterol in blood samples.^42^ When integrated with carbon nanostructures such as GO, CNTs their performance is significantly enhanced which enables to have highly selective and sensitive sensors for biomarker detection.^53–55^ It is worth noting that, tuning of the composition, crystal lattice structures, level of defects, electronic states enable control on the electroactive properties. Significant improvements in their performance have been achievable from the quantum confinement effect and electron transport systems.^56,57^ Nanoscale fabricated metallic oxide enables stronger analyte adsorption and thus provides an appropriate environment for biomolecules to have improved electron transfer kinetics.^58,59^ Sometimes, despite their narrow size range and broad range of selectivity, they can hardly meet the desired detection limit. Composites of different morphologies *e.g.*, nanotubular, nanorod, or as nanowire produced by template-based hydro- and solvothermal methods have been found with better catalytic activity compared to single component-metallic NPs or their oxides.^60–64^ Especially when metal NPs are allowed to deposit on metal oxides, the oxygen vacancies produced improve sensitivity for analyzing biomolecules like proteins, nucleic acids, and lipids.^65–67^ Accordingly, not only the detection limits are increased, but also broader sensing ranges are achieved in real samples.

**Fig. 5:**
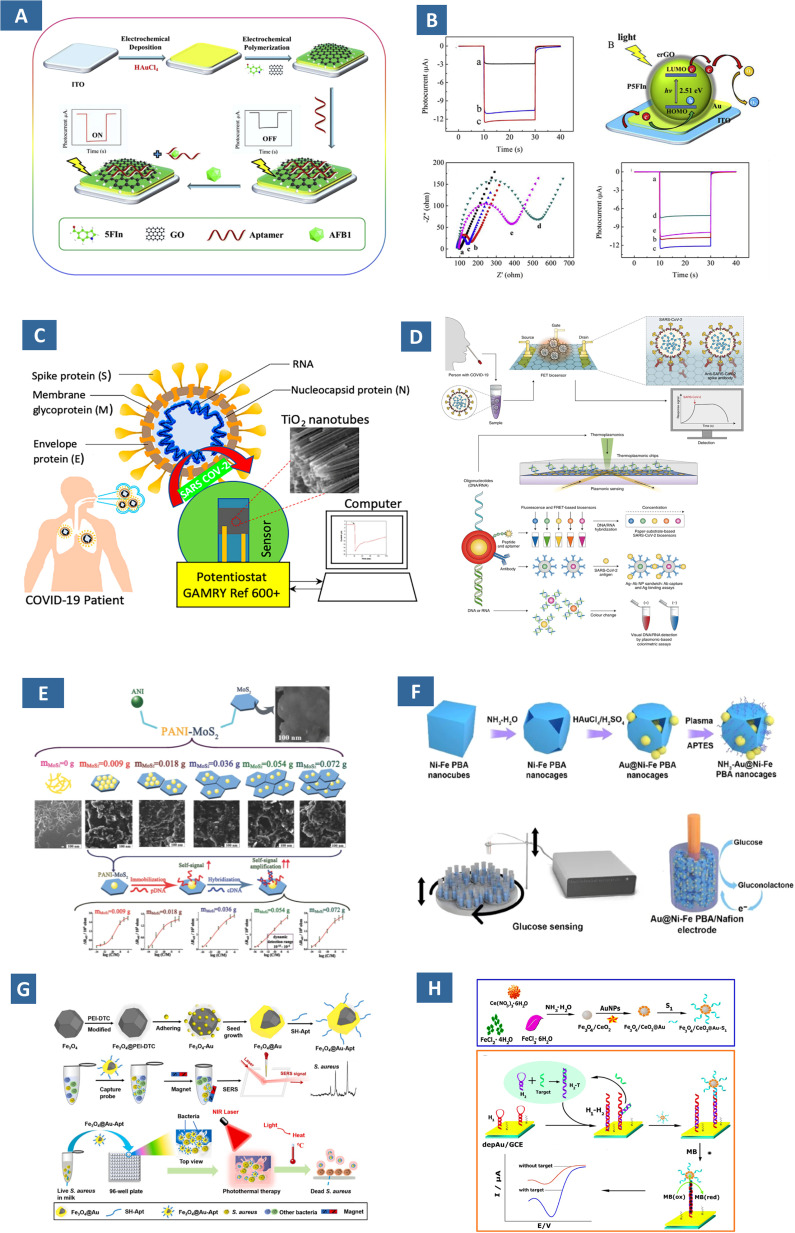
(A) Schematic of a reduced graphene oxide based photoelectrochemical aptasensor and (B) sensing response and its mechanism at various concentration of the modified ITO.^51^ (C) Biosensors for detection of SARS-CoV-2 fabricated from Co-functionalized TiO_2_ nanotube.^83^ (D) Two-dimensional gold nanoislands functionalized receptors in a dual-purpose biosensor for detection of SARS-CoV-2 by colorimetric and antigen-binding assay. Ab, antibody; Ag, antigen.^70^ (E) Effect of MoS_2_ on the sensitivity of PAni-MoS_2_ nanocomposites in detection of cauliflower mosaic virus.^84^ (F) Au@Ni–Fe embedded Prussian Blue Analogue nanocages for nonenzymatic detection of glucose.^85^ (G) Aptamer conjugated core shell NPs acting as nanoprobe for photothermal therapy of *S. aureus* and SERS detection.^86^ (H) Detection mechanism of microRNA-21 by Fe_3_O_4_/CeO_2_@Au–S1 composite in a biosensor.^87^

The combination of unique physicochemical properties, for instance, fluorescence quenching, UV absorption, photocatalytic, dielectric, and magnetic properties when leveraged in biosensors, they can be employed as target recognition elements. Among the techniques employed in biosensing using ZnO, TiO_2_, Fe_2_O_3_, CeO_2_, optical biosensing technologies are SPR, SERS, and surface-enhanced fluorescence (Fig. 5).^68,69^ For example, Au and Ag NPs bind to the SARS-CoV-2 viral antigens and generate detectable signals. Dually functional plasmonic NPs are integrated in such a diagnostic sensor with localized SPR sensing transduction. Complementary DNA receptors of SARS-CoV-2 nucleic acids remain in gold nanoislands (Fig. 5 (D)) and can precisely detect RdRp-COVID, F1ab-COVID and envelope (E) genes from SARS-CoV-2 from a multigene mixture.^70^ Keshavarz *et al.* revealed that the assembly of non-stoichiometric TiO_2_ and quantum scale arranged TiO_*x*_ can act as the recognizing unit for epidermal growth factor receptors for the diagnosis of breast cancer.^71^ The SERS scattering of TiO_2_ became more significant if oxygen vacancies are enhanced through the strategy. Reduction of size to quantum scale, substitution of metal cations with ions of different valence states could boost the enhancement factor up to 3.5 ×10^5^. As in the case of a flower-like gold modified NiO composite, doping of gold caused the nanocomposite to show enhanced performance in lactic acid detection.^72^ The homogeneous distribution of Au helps quick transduction of signals, while flower-like NiO nanostructures mimic enzyme activity towards the lactic acid oxidation. It has been reported to have a broad linear range of 100.0 M to 0.5 M, a high sensitivity of 8.0 A mM^−1^, and good selectivity against a variety of interfering biomolecules, such as uric acid, ascorbic acid, and cysteamine. Similarly, the highly required simultaneous sensing of acetylcholine and ascorbic acid has been described by mixed metal oxides of ZnO/CuO nanoleaves.^73^ Ternary metal oxide composites are reported in the work of Durai *et al.* and Alam *et al.* for sensing dopamine in biofluids.^74,75^ In the former, electrocatalytic active sites are offered by NiO, whereas ZnO and CoO in addition provided exceptional mechanical stability. The improved electrocatalytic activity of spinel nanostructures with different morphologies can be attributed to the environment they provide for enhanced electron transfer kinetics.^76–78^ Sensitive and specific detection of biomarkers are possible even by the detection of byproducts of such enzymatic reaction such as glucose oxidase and cholesterol oxidase, instead of immobilizing enzymes on electrode surfaces. Karuppasamy *et al.* described such a method to detect ascorbic acid in pharmaceutical samples with good anti-interference activity and a linear range of 5.0 M to 4.4 mM.^79^ Researcher developed a bimetallic copper oxide-gold nanoalloy for the colorimeter detection of glucose and H_2_O_2_.^80^ When CuO–Au nanoalloys were estimated for their colorimetric detection of glucose, the limit of detection (LOD) for the glucose was evaluated as 6.75 μM. The optimum condition for CuO–Au nanoalloys peroxidase-like activity at room temperature was also evaluated. A number of ceramic nanocomposites were employed as electrochemical immunosensor; Cu doped Al_2_O_3_ with graphite carbon nitride (g-C_3_N_4_) in the work of Miao *et al.* for the detection of amyloid β-protein (Aβ) and amperometric biosensor by Ouiram *et al.* for detection of choline. The later was assembled by immobilizing choline oxidase (ChO_*x*_), copper(i) oxide at manganese(iv) oxide (Cu_2_O@MnO_2_), and zirconium dioxide decorated gold NPs (ZrO_2_@Au NPs).^81,82^

There are cases when metal-based NPs react with analytes indiscriminately, leading to false-positive results. These are circumvented by their integration with specific biological recognition elements such as DNAs, antibodies, aptamers.^88,89^ In a previous study, enzyme free biosensor for detection of microRNA-21 involved the use Fe_3_O_4_/CeO_2_@Au magnetite NPs as a nanocatalyst. The catalytic hairpin assembly of the magnetite allowed the simultaneous hybridization of single and double stranded DNA for adsorbing electroactive species with highly amplified signals (Fig. 5(H)).^87^ Cobalt-functionalized TiO_2_ nanotubes (Co-TNTs) showed success in detecting the receptor-binding domain of SARS-CoV-2. The linear correlation across a broad concentration within around 30 s suggested its potential as POC diagnostics for SARS-CoV-2 detection from saliva samples and nasal secretions (Fig. 5(C)).^83^ A TiO_2_ NPs-based immunological assay based on PCR can quantify Salmonella in milk as low as 100 CFU mL^−1^, with high cell capture efficiency.^90^ The effects were crucial when ZnO NPs with unique electrical configurations were utilized prioritizing the interaction of NPs with the pathogen surface. Similarly, Fe_3_O_4_@Au nanocomposites modified with a suitable aptamer was designed for the SERS detection followed by photothermal therapy of *Staphylococcus aureus* and was shown in (Fig. 5 (G)).^86^ The method of analysis is not only simpler and faster than conventional procedures, but also can be used for eradication of pathogens to ensure minimal cytotoxicity. Molecularly Imprinted Polymer (MIPs), even though have been popular since their introduction with their recognitive features for particular biomarkers, their low conductivity and heterogeneous binding sites impose limitations in biosensing. This is possible to overcome when metal, metal oxide and their composites are used. Rezaei *et al.* developed a molecularly imprinted polysilicate on polyvinylpyrrolidone-stabilized gold NPs (AuNPs@PVP) synthesized by a water-compatible one-pot method.^91^ Using uric acid as the template, the resulting AuNPs@PVP@MIP was integrated onto a glassy carbon electrode, enabling sensitive electrochemical detection of uric acid in urine. On the similar note, pyrrole was electro-polymerized on an imprinted polymeric matrix consisting of Au NP functionalized Fe metal organic framework and nitrogen–sulfur co-doped graphene QDs for enhancing the electron transfer and binding channels for the polymer layer.^84,85^

Transition metal carbides, nitrides, and carbonitride, also termed as MXenes, were integrated with metal oxides nanocomposites for constructing sensors system. The self-assembling characteristics of MXene sheets aided in facilitating fast electron and ion transfer kinetics in enzymatic electrochemical sensors. Morever, they can act like quencher for fluorescence signals emitted by probes for detection of target molecules. Using FRET-based mechanism, Ti_3_C_2_ MXene nanosheets exhibited highly sensitive detection of exosomes in a wide range of concentration with a low detection limit of 1.4 × 10^3^ exosomes per mL.^92^ MXene nanosheets detect exosomes by using Cy3-labeled CD63 aptamer as a sensing probe. In the absence of the exosome, the fluorescence signal of the Cy3 was quenched by the MXene nanosheets. Stronger fluorescence emission signal can be achieved from MXene derived QDs which can be synthesized by acid exfoliation of MXene nanosheets.^93^ The change in intensity of the signal can be a basis to identify the interaction such as from the interference of any ion, charges on their surface. With the help of internal filtering effect, the researchers measured the concentration of Fe^3+^ in seawater and serum. The difference is that in the former the MXene nanosheet act like a quencher, whereas in the later, the fluorescent properties of MXene QDs itself get influenced.

The systematic combination of metal oxides with carbon-based materials, conducting polymers emerge as an interesting strategy to ensure bioanalyte immobilization. Reduced graphene oxide (rGO)/nickel oxide (NiO) nanocomposites were used on sensor electrodes to detect viral proteins and active influenza viruses (H1N1 and H5N2).^50^ The inhibition of signals by protein hemagglutinin (HA) while binding to electrode materials formed the basis of sensing. The performance of the created biosensors has the potential to detect influenza viruses and viral proteins with extreme sensitivity and selectivity. The composites show excellent photoelectrochemical properties for various chemical and biological analyses. Such a work was reported by Zhang *et al.* using rGO/poly(5-formylindole)/AuO (rGO/P5FIn/Au) nanocomposites. (Fig. 5(A and B)).^51^ In the aptasensor, electron–hole pairs are generated from P5FIn from irradiation of light, where noble metal/metal oxides amplify the signals and rGO embedded the AFB1 aptamer chain through π–π stacking interaction. The sensing range is wide enough (0.01–100 ng mL^−1^) despite the LOD of 0.02 pg mL^−1^. Transition metal dichalcogenide nanocomposites have layered structure analogous to graphene.^84,92–94^ Moreover, unlike graphene their bandgap is tunable with the addition of chalcogens and can be an essential means to employ them for detecting viral nucleic acid sequence. For instance, nanocomposite of MoS_2_ and, polyaniline (PAni) is used for sensing the existence of cauliflower mosaic virus 35S (CaMV35S) DNA (Fig. 5(E)).^84^ The electron rich surface of PAni allowed strong immobilization of probe DNA, accompanied by the increase in charge transfer resistance.

Electrochemical sensors when need to be integrated into garments or to be in contact with the surface of human skin, it is highly desirable from them to have sufficient flexibility to reduce motion-induced signal interference. A range of important biomarkers are found in human sweat, hence non-invasive detection of sweat metabolites is essential for acquiring biochemical information on human health status. While metal-based NMs have sensitivity, they are not flexible enough as much as they are in composites with the conductive polymer-based NMs. Xuesong *et al.* fabricated a sensing electrode by assembling PAni and polyurethane core–shell fiber. In the pH range of 2–7, the electrode demonstrated viability in sensing perspiration on the skin (sensitivity = 60 mV pH^−1^; capacity to detect pH change pH < 0.2).^95^ Multi-sensing platform has been developed to track metabolites (lactate) and electrolytes in saliva. Poly(3,4-ethylenedioxythiophene) (PEDOT)-modified graphene showed higher sensitivity and selectivity for detecting dopamine in a complex mixture.^94^ Zahed *et al.* developed a PEDOT-PSS modified 3-D stable porous, porous composite with Pt/Pd NPs to detect glucose and pH in human sweat.^96^

Metals and their oxides in the form of a thin-film electrode and aerogels are emerging as promising technology in wearable biosensors. Gold aerogels nanostructure, nanowires, and nanofibers modified wearable sensors have been found to detect glucose, lactates both quantitively and qualitatively in sweats. Gold nanowire aerogel and palladium aerogels showed superior sensitivity for detecting ethanol and glucose oxidase from sweat with sufficient stability.^97^ The electrochemical, colorimetric detection methods become limited when sample preparation is complex and requires high cost for POC testing. Solid-state thin-film transistors (TFTs) showed its promise with ease of miniaturization, robustness, intrinsic amplification, and rapid and label-free detection. The In_2_O_3_/ZnO heterojunction transistor was utilized to quickly detect 25(OH)Vitamin-D3 (Vit-D3) and uric acid (UA) in human saliva. The solution-processed In_2_O_3_/ZnO channel were functionalized with uricase enzyme; this facilitated strong coupling between the electrons in In_2_O_3_/ZnO heterointerface and the surface-immobilized bioreceptors and target analytes. Detection of biomolecules are often challenging due to small faradaic current and large IR drop, which has been compensated by fabrication of microelectrodes. Maduraiveeran and co-workers reported such microelectrodes made up of copper where uniform dispersion of gold occurs on copper oxide microflowers.^98^ The microsensor platform exhibited a broad detection range of 5.0 μM–0.5 mM with a sensitivity of *ca.* 4.14 mA μM^−1^ cm^−2^ and a LOD of *ca.* 1.41 μM for glucose and a linear range of 100 nM–88.0 μM with a sensitivity of *ca.* 6.19 mA μM^−1^ cm^−2^ and a LOD of *ca.* 27.0 nM.

### 5.2 Metal and metal oxide-based nanosponges: innovative sensing

Nanosponges (NSs) are hydrophilic, water-insoluble, and supramolecular three-dimensional (3D) hypereticulated polymeric nanoporous structures. The presence of lipophilic cavities and carbonate bridges produce hydrophilic channels which can offer improved solubility to both lipophilic and hydrophilic drugs. With a variety of attractive features, including minimal cytotoxicity and good biocompatibility, they are appropriate for biomedical applications due to their notable stability throughout a broad range of pH and temperature ranges. Polysaccharides specially those of starch derivatives are the good choice for NS materials; the various tunable functional groups make them interact with biological tissues strongly enough to promote bioadhesion. The first-generation NSs were synthesized using cyclodextrin crosslinked with carbonate, ester, ether, and urethane. The second-generation NSs were designed with specified characteristics such as fluorescence by introducing a charged terminal group, whereas in their next generation stimuli responsive properties are introduced. The sponges show structural variation depending on pH, temperature, or redox state at the drug delivery. Based on the type of crosslinkers, hydrophilic and hydrophobic sponges can be prepared.

**Fig. 6:**
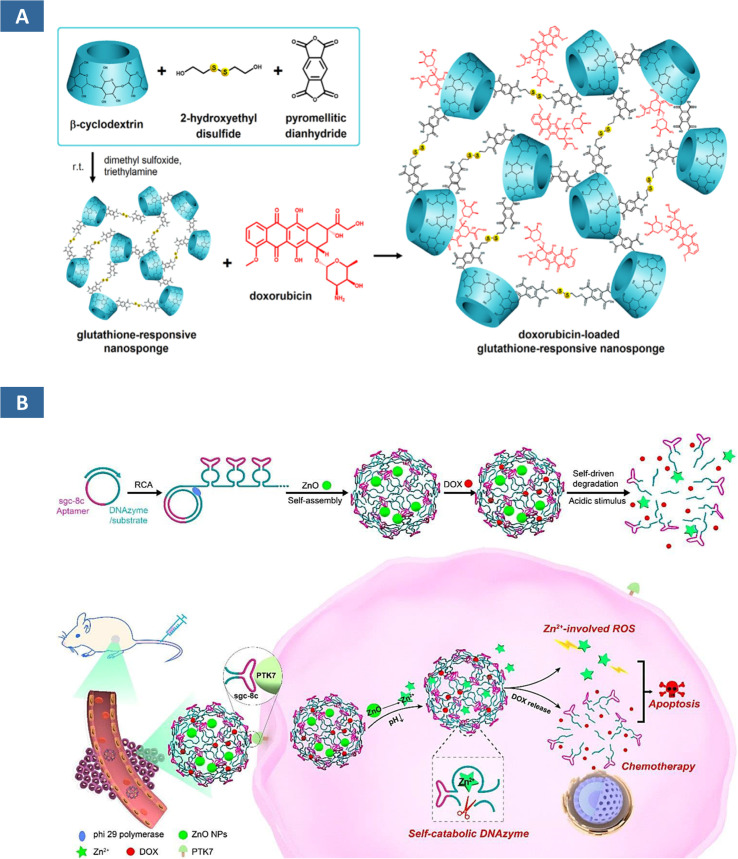
(A) Illustration of doxorubicin-loaded glutathione-responsive nanosponge.^104^ (B) ZnO-encapsulated deoxyribozyme nanosponges with multivalent tandem aptamer sequences for facilitated delivery of deoxyribozyme cofactors and therapeutic ROS generators.^111^

Cyclodextrins can be copolymerised directly with other monomers or grafted onto organic/inorganic compounds using their hydroxyl groups *via* a substitution/elimination process.^99^ The nature of the lipophilic cavities and a hydrophilic network depends on the type of cross-linkers used; hence NSs are ideal alternatives to improve the stability of volatile and insoluble compounds.^100^ As an antioxidant with therapeutic properties, water solubility of kynurenic acid has been improved through the use of cyclodextrin-based NSs (CDNSs).^101^ Accordingly, not only the solubility, higher drug-loading of about ∼19.06% and encapsulation proficiency ∼95.31% are achieved. In case of ferulic acid, the solubility has been possible to enhance up to 15-fold.^102^ The limited bioavailability of drugs has been addressed by using CDNSs. Their ability to form inclusion and non-inclusion complexes with various drugs is a result of their spongy morphology, which also makes them a useful therapeutic vehicle for the delivery of medications with low bioavailability. The cavity of CD permits hydrophobic molecules to be included, and the surrounding polymeric network can interact with less lipophilic molecules. The selective interaction is essential for delivering oral, and topical drugs, as well as targeted cancer therapy.^103^ Doxorubicin-loaded glutathione-responsive NSs are reported during assevesing the hepatotoxicity in human HepG2 cells *in vitro* (Fig. 6(A)).^104^ The NSs of polyvinyl alcohol and ethylcellulose synthesized through an emulsion solvent evaporation technique is deployed for targeted delivery of ribociclib, a metastatic breast cancer kinase inhibitor.^105^ Consequently, the drug release was highly accelerated with a maximum value of about ∼81.85 ± 0.37%. In addition, they demonstrated a greater degree of apoptosis in comparison to the free ribociclib, as well as strong cytotoxic effects against the breast malignant cell lines MDA-MB-231 and MCF-7. Such a selectivity towards drug encapsulation and delivery can be used for drug scavengers. One such use of β-cyclodextrin NSs (β CDNSs) are reported by Kumar *et al.* as reactive oxygen species scavenger.^106^ CDNSs crosslinked with 1-(3-dimethylaminopropyl)-3-ethylcarbodiimide hydrochloride upon modification with fluorescent carbon QDs (CQDs) and folic acid have been described in tumor theranostics.^107,108^ Efficient drug transport system has been developed from CDNSs functionalized with Au NPs with high loading capacities for phenylethylamine (90%) and 2-amino-4-(4-chlorophenyl)-thiazole (150%).^100^ The antiepileptic activity has been reported to be enhanced by neuropeptide-Y NSs in the work of Desai *et al.*^109^ Similarly, the anti-psoriatic potential of traditional hydrogel of Clobetasol propionate has been increased when they are loaded with NSs. The greater penetration of NSs to the epidermal layer is attributed to their higher anti-psoriatic activity.^110^ DNAzyme NSs showed more controllable drug delivery behaviour and suitable gene silencing functions. The DNAzyme NSs developed by Wang *et al.* contain an oligomerized DNAzyme-substrate framework which can capture ZnO depending on the pH of the environment (Fig. 6(B)).^111^ This tends to facilitate tandem aptamer arrangements for targeted delivery into cancerous cells. However, magnetic NSs are the recent additions in the realm of DNAzyme NSs. Tumour-targeted delivery is an essential aspect of such designed DNAzyme NSs. With appropriate environmental stimulation, DNAzyme causes cleavage of RNA by generated reactive oxygen species. These NSs had the ability to catalytically cleave HSP70 mRNAs, therefore downregulating protein expression and protecting cancer cells (MCF-7) against hyperthermia.^112^ They also suspect its potential in multimodal imaging of the cancer cells with higher permeability and retention effect.

NSs are proved as ideal RNA and drug delivery agents for infections by rhinovirus, respiratory syncytial virus, HBV, HSV, influenza virus, and human immunodeficiency virus (HIV). NSs with similar receptors like pulmonary type II epithelial cells are suitable attractant of SARS-CoV-2 virus.^113,114^ Incubating with NSs, the virus is neutralized and loses its potential instead of infecting healthy cells. Both of its forms, epithelial type II cell NS and human macrophage NS demonstrated their ability to neutralize SARS-CoV-2. NSs with cellular receptors have been claimed to effectively shield the human host cells from SARS-CoV-2 infection offering a basis of multifaceted antiviral strategy.^115,116^ Their presence reduced the interactions between the SARS-CoV-2, S-protein complex, and Human Angiotensin-Converting Enzyme 2 (ACE2), and in turn showed high affinity for binding to ACE2. There have been attempts to develop biocompatible NSs for usage against autoimmune antibodies instead of neutralizing exogenous poisons. Toxic chemicals are neutralized by NSs, which shield healthy cells from being affected. By incubating with red blood cell membrane-coated NSs, exogenous toxins might be completely neutralized. In order to achieve broad-spectrum neutralization, the NS platform differs from traditional NMs. As a result, it has been possible to treat consequences, especially when it's unclear which specific antigen triggers autoimmunity. A NS formulation has been used, which has showed great promise in treating antibody-mediated type II hypersensitivities in a way that is distinct from any other therapy. In contrast to current therapies, a NS formulation has demonstrated considerable potential for treating antibody-mediated type II hypersensitivities in the treatment of autoimmune haemolytic anaemia.^117^ The selective and specific interaction of NSs enable them to act as probe for analysing tablets and food sample. Nazerdeylami *et al.* showed that βCD NSs can detect diclofenac from its fluorescent intensity at 423 nm and can distinguish it from amphetamine, morphine, codeine, and ibuprofen.^118^

### 5.3 Graphene: a game-changing material for biosensing application

Among the two-dimensional NMs, graphene is an attractive choice as transducing material for ultra-sensitive biosensors in drug delivery,^119,120^ wearable devices,^121,122^ electronics,^123–125^ and bioimaging^126–128^ due to its multiverse interaction with biorecognition elements. The sp^2^ hybridized carbon atoms in their structure allow them to possess a single-atom layer thickness, high electron transfer rate, *i.e.*, good electrical conductivity and low electrical noise, high active surface area to modify when used as materials for bare electrode surfaces. A reason why they have been gaining attention in recent time among other biosensing materials is their easy synthesis process and patternable features as well as high functionality. Functionalized graphene has been integrated in biosensors for health monitoring and agriculture due to their easy synthesis, influence in electron transfer for developing sensitive biosensors.^129^ GO consists of functional groups such as –OH, carbonyl, epoxy, and –COOH groups on both the basal plane as well as at the edges of GO nanosheet. They are synthesized by oxidizing graphene, followed by dispersion and exfoliation in a solvent. The presence of such reactive oxygen functionalities permits the controlled nucleation and growth of metal, metal oxides, and semiconductor NPs. So far, their properties have been well explored for developing both optical and electrochemical sensors.

**Fig. 7:**
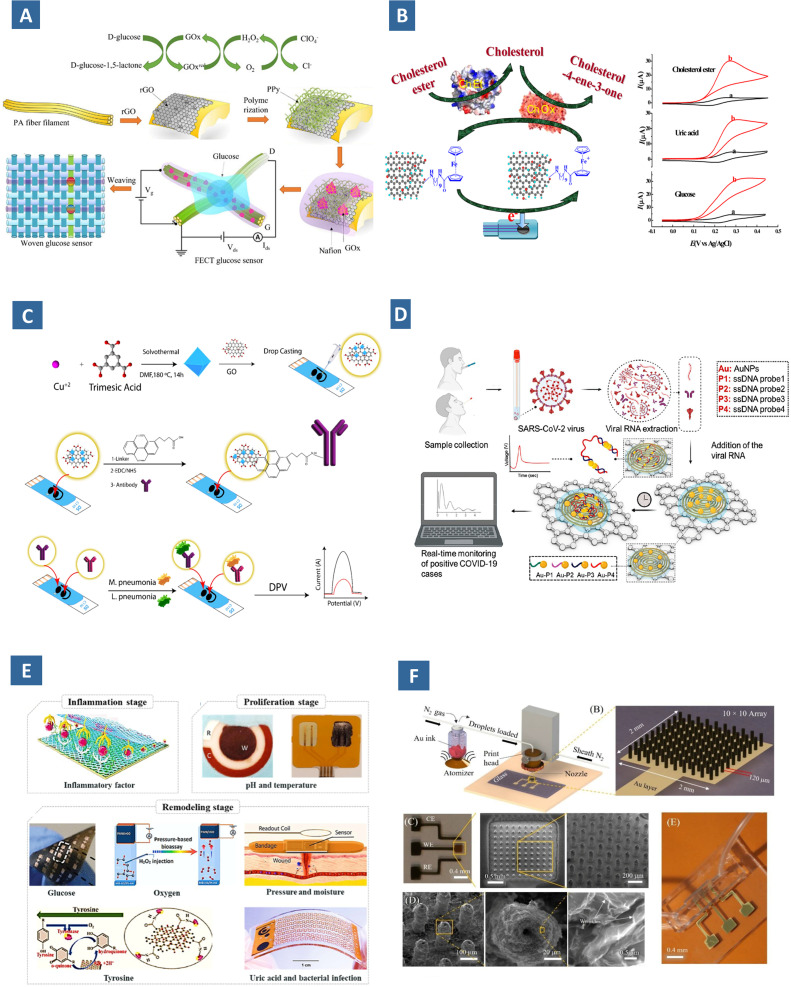
(A) Glucose sensing mechanism by a transistor-based glucose sensor with immobilized glucose oxidase-based on polypyrrole nanowire and rGO.^136^ (B) biosensing schemes of cholesterol ester mediated by functionalized graphene and the corresponding cyclic voltammetric response in (a) absence and (b) presence of the analytes.^137^ (C) Fabrication of GO/Cu–MOF composite based immunosensor for the simultaneous detection of M. pneumoniae and *L. pneumophila* antigens.^132^ (D) electrochemical sensor chip containing Au NP conjugated specific antisense DNA for the digital sensing of SARS-CoV-2 genetic content.^138–143^ (E) Graphene enabled wearable sensor for monitoring several biomarkers during wound inflammation, repair and remolding stage.^144^ (F) Graphene-based 3D printed sensor: schematic of the 3D NPs printer, printed gold pillar array and its SEM images, the micro-textured surface of Au decorated with rGO nanosheets, and complete device integrated into a microfluidic cell.^144^

Presence of oxygen functionalities on the surface of GO allow stronger and better adsorption of analytes. This makes them a potential antimicrobial NM for capturing multidrug resistant pathogens such as, *E. coli*, *Klebsiella pneumoniae* and *P. aeruginosa* for *in vivo* and *in vitro* studies, reported by Liu *et al.* and Tiwari *et al.*^130,131^ The dual detection of *M. pneumoniae* and *L. pneumophila* antigens has been manifested using a GO/Cu-MOF modified immunosensor utilizing the concept of differential pulse voltammetry (Fig. 7(C)).^132^ Morever, they act as fluorescence quencher due to the broad absorption and high mobility of electron. GO-based biosensor has been developed by Jung *et al.* where the adhesion of rotaviruses is detected from the quenching of photoluminescence of GO.^133^ Various types of electrochemical and optical biosensors based on GO typically include GO, rGO@polymer combinations, 3-D GO coordinated with different polymer hydrogel networks, GO@inorganic NP hybrids, and metal/metal oxide NPs with the purpose of detecting analytes, like H_2_O_2_, dopamine, UA, cholesterol, and glucose cofactors like nicotinamide adenine dinucleotide and adenosine triphosphate, cancer biomarkers and so on.^134,135^ These analytes can be detected by a number of approach. Nevertheless, among them surface functionalization is the most common. Surface functionalization is accomplished *via* covalent or non-covalent interactions, both of them have the similar purpose *i.e.*, to bring about the specific analyte to surface for binding interaction.

GO enhanced the electrochemical response by providing high number of enzyme binding sites, for instance in the composite of GO and chitosan, prepared through covalent modification of GO by a drop-casting approach.^145^ Covalent tethering of redox active units to the GO backbone through functional spacer can also enhance the ease in electron transfer. This has been demonstrated in an amperometric biosensors employed for quantifying cholesterol, uric acid, and glucose level in blood.^137^ The role of mediator ferrocene–GO couple is to influence the bioelectrocatalytic oxidation of the substrates in presence of cholesterol oxidase, uricase, and glucose oxidase respectively (Fig. 7(B)).^137^ While deposited on screen-printed electrodes, the superior reliability and efficiency of the composite was evident compared to its pristine counterparts. 1-Aminopyrene-rGO composite was prepared by Zhou *et al.*^146^ that showed enhanced performance similarly in detection of toxic phenols. The enzymatic receptor is immobilized on to the electrode materials through glutaraldehyde-mediated cross-linking. It showed high stability (retained >97% activity after 7 days of storage) and fast response time (<5 s), along with the LOD of about 2 and 7 μM for hydroquinone and catechol, respectively.

Graphene-based composites are integrated in immunosensors, biosensors that work based on antigen–antibody interaction. To detect biomarkers, bioreceptors like antibodies, aptamers, and enzymes are required to be bonded on the surface. EDC/NHS chemistry, where EDC refers to 1-ethyl-3-(3-dimethylaminopropyl)carbodiimide and NHS refers to *N*-hydroxysuccinimide, is the common approach for immobilizing antibodies and aptamers, and conversely physisorption for enzymes. Numerous oxygenated functional groups present in edge and basal plane of GO and rGO facilitate the effective immobilization of redox enzymes through either physical adsorption or covalent conjugation. Such application is first described in the work of Li *et al.*^147^ where graphene–PAni and carboxylated GO have been used in the transducer to detect estradiol in water and milk samples. The carboxylated GO facilitated the formation of horseradish peroxidase–GO–antibody conjugates to bind with 17β-estradiol. The performance was better than conventional competitive electrochemical immunosensors and aptamer-based electrochemical sensors.^148,149^ GO based immunosensor was also developed by Ge *et al.* and Ali *et al.* to immobilize monoclonal antibody and anti-apolipoprotein B-100 for the detection of cancer antigen 153 and LDL cholesterol respectively.^150,151^ Both the sensor showed high loading capacity of the antibody, fast reaction time and stability. GO was modified using click chemistry. In the former, the composite was synthesized from azide-functionalized magnetic silica NPs and acetylene-functionalized GO through metal catalysed 1,3-dipolar cyclo-addition reaction. Apart from the ease of heterogeneous electron transfer in rGO, electron conduction is also found to be favourable in the composite of metal-rGO.^152,153^ The higher electrical conductivity of Au NPs-rGO composite for the electrochemical detection of myoglobin is attributed to the inter-penetrating network formed by Au NPs.

The properties of GO and its derivatives have been found to be essential for label-free optical sensors such as optical fiber-based sensors, SPR sensing, and SERS for antibacterial activity and bioimaging in numerous studies. A range of optical biosensors have been developed leveraging them to sense a variety of biomolecules, including antigen antibodies, nucleic acids, proteins. The sensors tend to operate based on distinct biomolecular interactions with the benefits of rapid detection and high sensitivity. For the purpose of detecting human T lymphoblastic cells, Tan and coworkers have produced a GO-aptamer combination (GO-apt). Along with a limit of 10 cells per mL, they were able to quantify the cells across a wide spectrum, including 1 × 10^2^–1 × 10^7^ cells per mL, based on the fluctuations in fluorescence signals.^153^ An N-FK51A glass prism-based SPR sensor covered with graphene was the subject of a thorough analytical investigation by Panda *et al.* to detect glucose in human blood serum (25–175 mg dL^−1^) efficiently.^154^ Graphene and Au layer thickness can be changed to maximize performance of the sensor at 589 nm. GO and its derivative can play role as a quencher of fluorescence of labelled DNA samples when they get adsorbed. GO while functionalized with bismuth ferrite showed photoactive characteristics with accelerated charge transfer ability. Zhou and coworkers integrated them with an anchor DNA-conjugated magnetic bead, and prostate-specific antigen (PSA) aptamer/trigger DNA.^145^ When interaction occurs between the target PSA and the aptamer, H_2_O_2_ produced by the consecutive DNA chain hybridization events consumed photo-excited electrons from rGO-BiFeO_3_ when exposed to visible light, hence increasing the photocurrent. The enzyme-catalyzed photoelectric reaction enabled the biosensor to detect PSA up to about 0.31 pg mL^−1^ in phosphate buffer saline as well as in human serum.

Selective attachment of the rotavirus target cells is guaranteed by the highly selective interaction between Au NPs and the amino functional groups of the DNA nucleotides. Since they are highly reproducible, inexpensive, and quick to diagnose, GO-based microfluidic immunosensors are becoming viable substitutes for conventional pathogen-detection methods like cell culture and rt-PCR. The 3D nanoprinted immunosensor known as the ‘3D-printed COVID-19 test chip’ were coated with nanoflakes of rGO.^144^ This device was created from a micropillar array coated with rGO nanoflakes and functionalized with spike S1 antigens of SARS-CoV-2 by layer-by-layer printing (Fig. 7(F)).^144^ SEM images show micro-textures of printed micropillar array. Au NPs conjugated with a specific antisense oligonucleotide, can be allowed to aim at virus nucleocapsids of SARS-CoV-2.^138–143,155,156^ As shown in Fig. 7(D),^138–143^ In the presence of a target viral RNA, significantly improved signal can be obtained within 5 min of incubation, with an LOD of 6.9 copies per μL and a sensitivity value of 231 copies per μL with-out the need for further amplification. Even maximal sensitivity and specificity have been reported where the sensor distinguishes negative samples from the positive. Natural bioreceptors may not always be as stable as they could be, but synthetic biological molecules that resemble receptors, such as molecularly imprinted polymers (MIPs), may be utilized as their substitute. Through the process of molecular imprinting, polymerization leads to the formation of cavities complementary in dimensions and chemical functionality to the target molecule. A number of hybrid structures are produced by integrating them with GO and their derivatives. In the composites, the role of GO can be both *i.e.*, ensuring stronger adhesion surface as well as a conductive nanofiller. Their efficiency can be understood from robust binding of analytes at ultralow concentration.^157^ These optical, electrochemical, and piezoelectric GO-based sensors laid out the foundation of cost-effective POC diagnostic and lab-on-a-chip systems. Goyal and coworkers stated in their findings that, molecularly imprinted polypyrrole (PPy) doped with hexacyanoferrate and coupled with β-cyclodextrin-functionalized reduced graphene oxide can be employed to detect stress biomarkers.^158^ The inclusion site provided byβ-cyclodextrin enhances specificity while hexacyanoferrate act as a simple redox probe. Dual MIP 3-nitrotyrosine and 4-nitroquinolin-*N*-oxide while coated on GO QD improves the electrode surface area which allows to have highly amplified electrochemical signal.^159^

Despite the fact that, pristine GO show excellent fluorescence quenching properties, metal NPs, QDs show size dependent fluorescence and electrocatalytic properties, which paves the way of fabricating GO-NP or QD decorated sensors for detecting and quantifying metabolites. For example, Pt NPs-CQD/ionic liquid-functionalized GO nanocomposites are fabricated by Chen *et al.* for detecting H_2_O_2_.^160^ The high abundant active sites improved the electrocatalytic reduction of H_2_O_2_, in a broad linear range of 1 to 900 mM and detection limit of 0.1 mM. A novel poly(ionic liquids) functionalized polypyrrole–GO nanosheets possess improved transmission mode of electrons and assist in the oxidation of dopamine.^161^ Neurological illnesses like schizophrenia and Parkinson's disease have been linked to malfunction of dopamine, one of the most significant neurotransmitters. Conducting polymers allow the conjugation of biomolecules with more stability. PEDOT–GO is an example of this type of composite utilized for the same purpose.^162^ Both of the cases, the electron transfer kinetics are improved by functionalizing the surface with conducting metal NPs and conducting polymers. The ZnO nanorod-based enzymatic glucose sensor showed better performance while incorporated with rGO under UV irradiation as reported in the work of Zhou *et al.*^163^ In addition to facilitating electron transport, the inclusion also inhibits the rapid recombination of photogenerated electrons and holes. The stimulation of the holes in the valence band of ZnO nanorods increases the catalytic activity of glucose oxidase towards glucose.^164,165^ In presence of UV irradiation, an increased sensitivity of 1.7-fold has been observed for rGO based composite, despite lowered detection limit of about 2-fold. In another composite sensor of rGO and polypyrrole improved electrical characteristics of fibre transistors have been achieved (Fig. 7(A)).^136^

Another prevalent use of graphene is fabricating wearable sensor for remote sensing and real time monitoring of bioanalytes, which stems from its excellent mechanical strength (Young's modulus 1.0 TPa, tensile strength 130 GPa).^136^ Wearable sensing materials from the graphene family are integrated with a variety of flexible substrates *e.g.*, PI, polyethylene terephthalate, PVC, PDMS, silk fibroin, cellulose, SiO_2_, and Al_2_O_3_.^166,167^ Wearable sensors monitor various components in body fluid; for instance, in sweat pH, ion, pyruvate, tyrosine, lactic acid, cytokines, cortisol, uric acid, and glucose. They are also associated with wound healing processes (Fig. 7(E)).^144^ Dynamic analysis of human sweat cortisol has been possible.^117^ In harsh aquatic settings, including swimming or diving, waterproof and microfluidic sweat patch allowed for sweat collection and *in situ* biomarker analysis.^168^ Moreover, the abundant oxygen-containing functional groups endow it to have excellent water solubility in physiological environment. In order to avoid non-specific protein adsorption and bacterial contamination, polymers both natural and synthetic are used to modify GO. At present, GO is integrated in electrochemical and optofluidic microfluidic chips with a target of multiplex sensing. Such a microfluidic platform has been proposed by Trau *et al.*^169^ that uses GO and Au-based biochips for sensitive cell and protein analysis in human serum samples. Higher capture yields achieved by the chips become possible due to oxygen functionalities and aromatic ring structures, which is first time to be reported in literature.^170^

## 6. Paper-based biosensors

Paper substrates have great features compared to other diagnostic devices, *e.g.*, simplicity of fabrication, biological compatibility, and portability and easy disposal. They serve a greater interest in accurate, and cost-effective diagnosis. Papers specially those of cellulose fibre content-based or membranes made from nitrocellulose are used in such biosensors which operate based on colorimetric and electrochemical techniques. Colorimetric techniques involve use of a dye or indicator which produce distinct response in presence of an analyte, while electrochemical methods measure the current when a reaction occur between the analyte and a working electrode. Especially, proteins with their high sensitivity and abundance produce certain immunoreactions. In addition, the paper substrates can produce flow due to their inherent capillarity properties. These sensors are more user-friendly, a common example maybe the pregnancy test that takes only a few minutes to detect human chorionic gonadotropin hormone released with urine. A variety of paper-based biosensors are reported to date, highlighting one or more factors for the quick diagnosis such as the method of fabricating the device, the type of analyte it will detect, the technique of detection, and the applications that were demonstrated. For example, colorimetric sensors even though are more user-friendly, they have limitations due to their sensitivity in coloured matrices. Contrary to it, electrochemical methods offer higher sensitivity with their more intricated arrangement that sometimes they are appropriate for quantitative detection in affordable options.

Electrochemical detection with paper substrates has been recently found promising, generating high-precision electrochemical paper-based analytical devices (ePADs) for point-of-need applications. They are usually fabricated through stencil, screen-printing, and laser scribing for producing conductive ink layer on the substrate. The surface of the substrate is usually modified by incorporating conductive NMs such as CNTs or Au NPs. They serve as covalent anchoring points for the biorecognition probes in addition to facilitating the rapid flow of electrons between the transducer and analyte. They not only provide for rapid transfer of electrons between the transducer and analyte, but they also serve as covalent anchoring points for biorecognition probes. MWCNT-Nafion was deposited on chromatography paper by Valentine *et al.* to correlate the pore size of the papers with MWCNT network during electrochemical sensing of glucose.^170^ Au doped SWCNT electrodes were fabricated onto a nitrocellulose membrane according to the report by Tran *et al.*^171^ Along with notable conductive properties, remarkable mechanical durability, they showed their potential to be developed as non-enzymatic glucose ePADs. Apart from glucose, some other most relevant biomolecules like H_2_O_2_, dopamine, urea, and cholesterol are monitored using wearable paper-based electrochemical biosensors. They have a propitious framework for POC testing owing to their high adaptability, and inherent self-pumping capability. For example, Mazzaracchio *et al.* created a paper-based electrochemical biosensors for the detection of iron ions in serum samples using carbon black complementary metal oxide semiconductor and Au NPs with a high detection of 0.05 mg L^−1^ and a linear range of values of 10 mg L^−1^.^172^ Glucose detection PAD by Yamaoka *et al.* constituted from a complementary metal-oxide semiconductor chip and fluidic channels.^173^ The two-layered channel with filter layer (for filtering a sample) and an enzyme layer (for reacting with an enzyme), the sensor showed capacity to measure glucose levels from 0.5 to 10 mM in human blood serums. When compared to traditional electrochemical biosensors, the use of printed electrodes on paper-based electrochemical biosensors offers several significant advantages including ease of use, cost, and disposability. ePADs generally employ an immobilized aptamer covalently or noncovalently bonded with the substrate to interact with the target receptors of the antibody or cell. By incorporating nanocomposites typically, the incubation procedures and the flow of sample. Srisomwat *et al.* reported the procedure of label-free detection of hepatitis B virus DNA using acpcPNA.^174^ Immobilization of aptamers to GO/SiO_2_ nanocomposite enable sensing of Dengue virus strands with wide range of serotypes.^175^ In another study, Cinti *et al.* immobilized aptamers tagged with methylene blue on Au NPs by drop-casting method to develop a HIV virus multiplexed sensing device.^176^ The improved charge transfer kinetics at the electrode surface is reflected from their short incubation time with detection of double-stranded HIV DNA in serum samples in the linear range of 3–3000 mM. The sensitivity of microstructured-gold ePAD biosensor was attempted to enhance using Au NPs modified with single-stranded antisense oligonucleotides (ssDNA) for the identification of SARS-CoV-2 nucleocapsid phosphoprotein RNA.^177^ The ssDNA-conjugated Au NPs selectively interact despite of genomic mutation without the complicacy of gene amplification step required in the conventional RT-PCR. The biosensor successfully recognized COVID-19 in positive (symptomatic and asymptomatic) and negative patients by the nasopharyngeal sample assay along with distinguishing SARS-CoV-2 from SARS-CoV and MERS-CoV (Fig. 7(D)).^138^

**Fig. 8:**
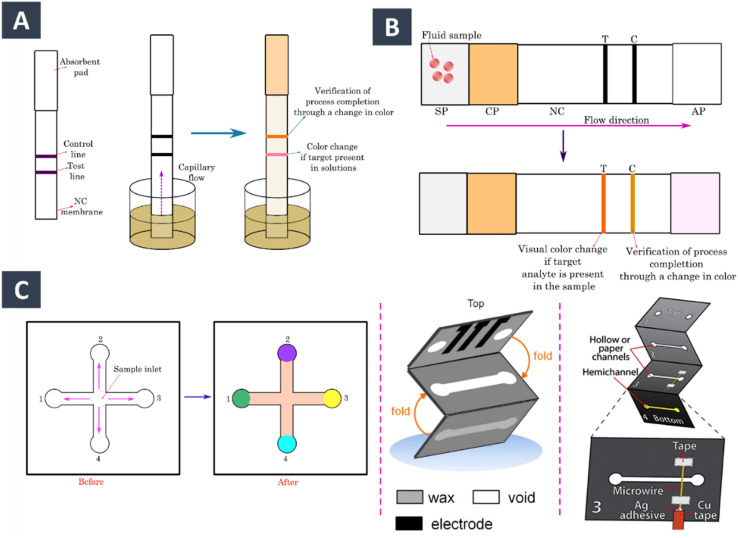
Schematic illustration of different paper-based assay for biosensing (A) dipsticks comprises of a test line and control line printed on nitrocellulose membrane (B) SP: sample pad, CP: conjugate pad, T: test line, C: control line, and AP: absorbent pad in a lateral flow assays (C) microfluidic device for detecting multiple analytes (left) and an origami-based 3D μPADs.^183,184^

Among the paper-based biosensors, dipstick assays, microfluidic paper analysers (μPADs), and lateral flow assays (LFA) are some of the commonly used commercially available paper-based diagnostics tools. Exosomes are one kind of extracellular transport vehicles released by cells. Especially those released by tumour or cancer cell are abundant in body fluids; hence they can be effective as diagnostic and prognostic biomarkers for various malignancies.^178^ LFA, an advanced platform involving multistep processes (*e.g.*, extraction, separation, and chemical transformation) (Fig. 8(B)). Au NPs and QDs-modified SiO_2_ core colorimetric and fluorescent dual-functional LFA has been employed for COVID-19 diagnostics.^179,180^ Colorimetric LFIA biosensors have been developed by Rodríguez *et al.* for the accurate quantification of exosomes from a human metastatic melanoma cell line.^181^ In this study, exosomes are embedded in the mixture of anti-CD9 and anti-CD81 for visualization of exosomes from different sources and Au NP-conjugated anti-CD63 as detection antibody. Yu and colleagues constructed an LFIA to find exosomes derived from non-small cell lung cancer.^182^

**Fig. 9:**
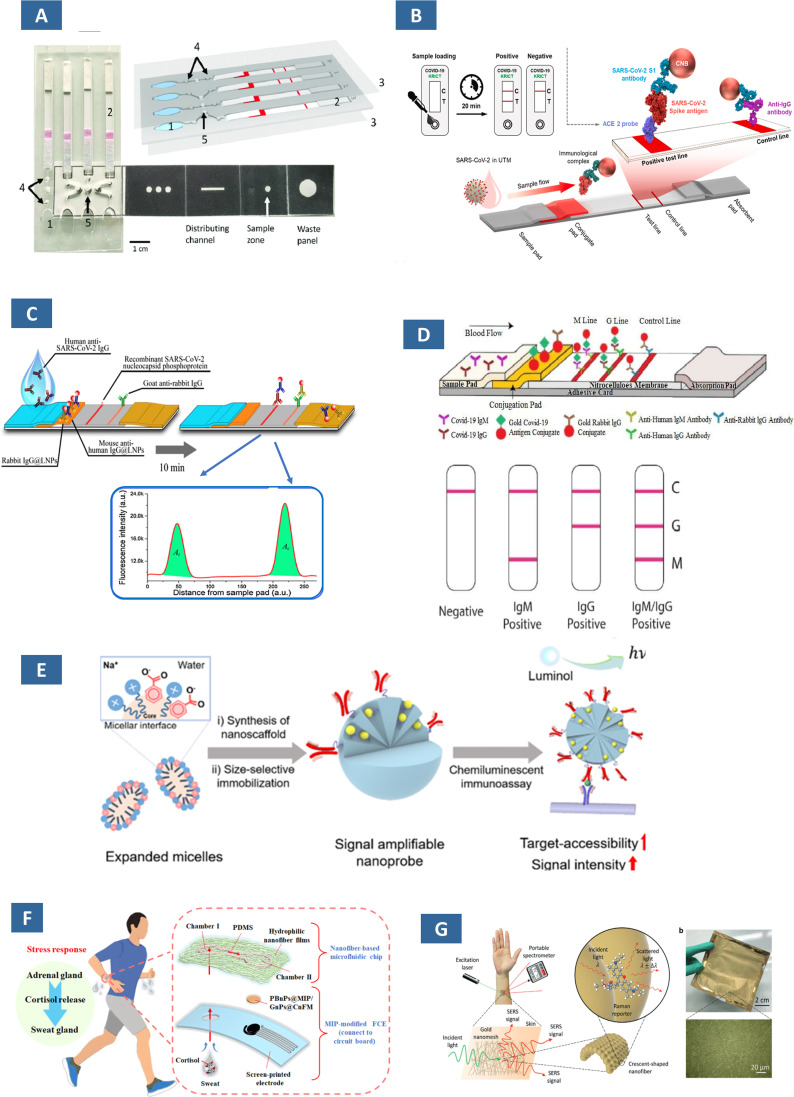
(A) Loop-mediated isothermal amplification-based DNA detection by a paper-based microfluidic device attached with a foldable paper strip. The dark regions of paper strip contain hydrophobic wax whereas the unpattern regions allows direct sample flow.^151^ (B) Schematic of the recognition of SARS-CoV-2 spike antigen by angiotensin-converting enzyme 2 cellular membrane receptor using LFIA assay.^189^ (C) Fluorescent LFIA assay for detection of anti-SARV-CoV-2 IgG in human serum.^187^ (D) IgM–IgG antibody test kit for SARS-CoV-2 detection: illustration of the LFIA device and interpretation of the results obtained from the device where C: control line, G: IgG line, M: IgM line.^186^ (E) Mechanistic scheme of detection of avian influenza viruses (AIVs) using a nanoprobe-based chemiluminescent LFA.^194^ (F) Real time cortisol monitoring with the help of a nanofiber-based microfluidic chip integrated in molecular imprinted electrochemical sensor.^192^ (G) Gold nanomesh based wearable SERS sensor designed for detecting biomarkers in sweat (optical microscopy image of the gold nanomesh has been shown in inset).^197^

Au NPs functionalized with CD63 aptamer were used as the recognition element; the probe was successful to isolate A549 exosomes from human lung carcinoma cells in an amount of about 6.4 × 10^9^ particles per mL. Au NPs in their pristine and functionalized forms are extensively used in LFA type assays for antigen, antibody detection of SARS-CoV-2. Nevertheless, the high sensitivity and specificity, very often the cross-reactivity and genetic mutation of SARS-CoV-2 resulted in false positive results. Recently, rapid antibody detection has been possible using Au NPs conjugated with mouse anti-human IgG.^185^ The sensitivity of this assay was further improved in an almost similar attempt of Li *et al.* who conceptualized the simultaneous detection of IgG and IgM antibodies.^186^ Illustrated in Fig. 9(D), nitrocellulose membrane in LFA kit contains respective antigens to bind with the IgM and IgG antibody found in Covid-19 affected patients' blood serum.^186^ Such detection measures can also be performed following the principles of fluorescence, luminescence, and surface plasmon phenomena. The manifestation has been found in the work of Chen and coworkers with LFIA assay using lanthanide-modified polystyrene NPs (Fig. 9(C)).^187^ In order to detect SARS-CoV-2 S antigen nanozyme (Co–Fe@hemin-peroxidase) were incorporated with LFA to achieve sensitivity analogous to enzyme-linked immunosorbent assays (ELISA) method.^188^ ACE2, in presence of cellular receptor prompt detection has been possible (Fig. 9(B)).^189^ Lateral flow biosensors (LFBs) are another novel paper-based device that works combinedly on the basis of immunochemical reactions and chromatography.^190^ As discussed before, like LFIA this also involves NPs-labeled protein–target complexes in a sandwich format comprising the test and control areas, respectively. Accumulated NPs in the test section produce distinct colors depending on the interaction with the analyte. The test is conducted on “lock and key” concept of antigen–antibody interaction. Au NPs and QDs are included in this LFB strips to improve the assay sensitivity. Dual Au NPs based LFB enable molecular profiling of exosomes from MCF-7 cells with a LOD of 1.3 × 10^3^ particles per μL using CD9/MIC3 antibodies.^191^ This is about more than 12-fold compared with conventional LFA. On the other hand, μPADs are cellulose based kits for analysing complex biological samples such as proteins, nucleic acids, toxins (Fig. 9(C)).^187^ Due to their small volume, they do not require any external support to move through body fluid. The liquid sample containing the analyte is allowed to move through the strip with the aid of capillary action associated with molecules. Wearable paper based electrochemical sensors are fabricated from nanofibers and a molecular imprinted polymer (MIP) through microfluidic technique for sweat analysis *in vivo* and *in vitro*.^192^ The sensor constituted of a MIP-modified electrode for sensing and a nanofiber-based microfluidic layer for spontaneous sweat pumping (Fig. 9(F)).^192^ Nanoprobe based chemiluminescent LFA biosensors have been developed for diagnosing diseases caused by avian influenza viruses (AIVs). An origami paper-based microfluidic device has been constructed by hot wax printing that either repel or attract blood during detection of DNA specific to malaria.^193^ The device is proved to be highly sensitive and specific while detecting malaria even its several strains (including *P. vivax*, *P. malariae*, *P. ovale* and *P. falciparum*) after only 45 minutes of amplification (Fig. 9(A)).^193^ Jung *et al.* used the probes for selective immobilization of antibodies and enzymes; for low pathogenicity avian influenza H9N2, H1N1, and detection limits of high pathogenicity avian influenza H5N9 viruses are achieved that is significantly lower than those of commercial rapid test kits (Fig. 9(E)).^194^ With the purpose of detecting antibodies (CR3022) of SARS-CoV-2, an impedimetric immune μPAD was fabricated on chromatography paper using screen-printed working electrode of ZnO nanowires.^195^ ZnO NWs produced sensible response with considerable charge resistance change despite their variation in surface area. Such a detection measures provided smaller detection limit, and faster assays even than ELISA kit. Using a portable sandwich assay with QDs, Li *et al.* developed nitrated ceruloplasmin, a significant biomarker for cardiovascular disease, lung cancer, and stress responses to smoking.^196^ The assay showed detection limit up to 8 ng mL^−1^ in human plasma sample. The sensor has also been used for rapid detection of prostate specific antigen (PSA) in human serum. Anti-PSA coated CdSe/ZnS NPs when fused with the immunochromatographic strip with the electrochemical detector, quantitative signal transduction has been possible. Sensitive detection with a limit of 0.02 ng mL^−1^ PSA in human serum is achieved.

Paper-based fluorescence biosensors include fluorophores; fluorescent proteins embedded on the strip to interact with a ligand that act as quencher. For example, fluorescently labeled anti-CD63 immobilized on MoS_2_-MWCNTs served as fluorescence quenching agents for PE-conjugated antibody, an exosome generated by breast cancer cells.^198^ Application of fluorescent nanosphere has also been observed in LFAs with a membrane biotynylation technique where the lipid membrane of exosomes is modified with biotin modified with biotin, to have stronger interaction with antibody such as those reported at a rate of 85%.^199^ p-ELISA provides faster detection with lower sample volumes retaining the same sensitivity and specificity offered by conventional techniques. Such as those developed by Lee *et al.* allowed targeted detection of exosomes using streptavidin agarose resin-based immobilization.^200^ They studied the systems with and without CD63 captured antibody immobilization, fetal bovine serum, and EVs/exosome-depleted fetal bovine serum.

## 7. Important application of nanosensors in healthcare

### 7.1 Enhancing communication and networking systems

Nanotechnology has taken a pivotal role in prevention and control of diseases measures apart from their detection. The accuracy of measurements of such sensors not only alleviates the limitations imposed by the microscaled devices, but also holds promise for next generation targeted delivery. Implantable nanosensors now save lives of patients of chronic diseases through continuous monitoring. Nanosensors albeit employ NMs as its core component, in order to completely utilize its potential, it is often required to deploy them in denser network structures. The terahertz range operation frequency of the sensors causes high attenuation. As for many instances, their application is limited for oversimplified architecture and high-power consumption accompanied by high path loss and scattering effects. Integrating them in highly concentrated arrangements within interconnected settings is what we call nano-sensor networks or nano-networks. Sensors are employed to do both intervene with cells directly or indirectly to exchange information.

Several approaches are practiced for nano-machines to communicate with one another and can be outlined as – molecular, electromagnetic, acoustic, and nanomechanical. Among them, molecular and electromagnetic communications have emerged as prominent wireless technologies facilitating intercommunication. The former entitles the transmission and reception of information encoded in molecules while the electromagnetic communication is the transmission and reception of electromagnetic radiation from components based on various NM. The use of terahertz band frequency reduces the high complexity and energy requirements of miniaturized sensors due to its low signal attenuation and high bandwidth. Current researches are devoted to overcome the loss of signals by proposing innovative transceiver design and novel network-layer methods. Miniaturized transistors like FinFET and 3D Tri-gate transistor successfully overcome the limitation of power loss.^201^ Graphene based multiple input multiple output antenna system serves as a nano-patch antenna that can be programmed dynamically according to the need for efficient intra body communication.^202,203^ Unlike this, molecular communication entails the release of a nanotransmitter molecules in biofluids along with the subsequent propagation to the receiver. The nano-receiver then detects the message encoded in them in terms of different properties such as concentration, type, release timing, *etc.* Neuro-spike communication, one of the most evolved concepts of molecular communication, works on passing electrical impulses among the neurons. Single-input single-output and multiple-input single-output are two such physical models for this communication.^204,205^ Nanoscalic events like FRET can also be exploited to develop molecular communication method between functional fluorophores. In presence of relay fluorophores, longer excitation signal can enable communication between sender and receiver located 200 nm apart.^206^

The abundance of the nanosensors with their feasibility to connect with IoT shifted the communication to a new paradigm, called Internet of Nano Things (IoNT). IoNT involves components like nanoprocessors, nanobots, nanoclocks, and nanomachines that together can provide continuous health monitoring and personalized treatment plans. Intra-body nanosensor enables real-time monitoring of vital signs and early detection of health issues. Along with the communication within the human body (intra-body), transmission of data from internal sensors to external devices is also possible. Nanorouters and other nanofabricated devices can be directly communicated through radio frequency messaging by interconnected nanonodes with the use of simple commands. Body area network consists of interconnected sensors and actuators operating in and around the human body. The collective physiological data such as heartbeat, insulin concentration, blood pressure can be monitored and taken the appropriate measures before any catastrophic consequence takes place. The better and early anticipation of diseases is important to improve the life expectancy and management of chronic diseases.^207^ The data generated by nanosensors can be analysed using big data analytics to gain insights into patient health and improve medical decision-making. This helps in telemedicine, precision medicine, predictive, preventive, personalized, and participatory healthcare.^208^

### 7.2 Precision and early detection of disease

Early and accurate detection of diseases is the key for designing a précised therapeutic regimen. Such challenges are met through biomarkers analysis. The example includes nanosensors integrated into lab-on-a-chip devices for point-of-care testing. Nanosensors are highly sensitive and miniature devices that operate at the nanoscale to detect and measure physical, chemical, and biological changes. These sensors provide high sensitivity and specificity, rapid real-time or near-real-time response, miniaturization to facilitates integration into portable or implantable devices and cost effectiveness to potentially reduce the cost of diagnostics and treatment. Acting at the molecular and cellular levels, these nanosensors have greatly enhanced the prospects for diagnosis and programmed treatment. These derive their impressive sensitivity and specificity from a few properties that come with high surface-area-to-volume ratios. Nanosensors consist of a transducer, which can be optical, electrochemical, or magnetic, that converts the interaction of recognition elements with specific target molecules or analytes into a quantifiable signal. Nanosensors have become powerful tools for both clinical and scientific purposes, allowing for incredible ends mainly because of the use of nanostructured materials such as metal or polymer NPs, CNTs, thin films, and nanoscale wires.^209^

Unlike traditional diagnostic techniques, nanosensors are vital for point-of-care systems and play a critical role in the bioanalytical efficiency of a test. Nanosensors are increasingly used in many disease areas, including COVID-19, HIV, hepatitis, stroke, diabetes, and obesity. These nanosensors are important point-of-care diagnostic (POCD) tools, that have a lot of potential in personalized medicine for the detection of disease markers in body fluids by observing biomarker signals or chemical reactions. Because of their high reactivity and selectivity, these sensors allow rapid disease evaluation and treatment.^210^ Further integration of nanosensors with microfluidics and cutting-edge imaging technologies further improves their diagnostic capabilities enabling early diagnosis and remarkably accurate disease progression tracking.^211^

One of the innovative applications of nanosensors is the detection of biomarkers associated with bacterial and viral diseases. In cancer diagnostics, nanosensors can detect tumour-specific proteins or nucleic acids in extremely small amounts, enabling early intervention and better prognosis. The rapid and accurate detection of viral antigens and antibodies in infectious diseases like COVID-19 has been made possible by nanosensors. Electrochemical nanosensors integrated with wearable technology enable real-time glucose monitoring, providing patients health information with metabolic illnesses such as diabetes with greater control. Their proposed use in nano fluidics, which encourages the use of nanosensors platforms to increase diagnostic accuracy in the identification of incurable diseases like diabetes and cancer, further emphasizes the promise of nanosensors networks.^212^

Even with their great potential, manufacturing scalability, cost, and integration with the current healthcare infrastructure remain obstacles to the broad use of nanosensors. Two important issues are ensuring biocompatibility and reducing the possible toxicity of NMs. These issues should be resolved in the future by developments in data integration, fabrication methods, and materials science. Their usefulness will further increase by creation of multifunctional nanosensors that can detect several analytes at once. Predictive analytics and increased diagnostic accuracy are anticipated when artificial intelligence and machine learning are integrated into nanosensors platforms. They offer early, precise, and non-invasive diagnostics, thus have become revolutionary tools in the detection and treatment of diseases. Their entire potential will be revealed by ongoing research and development, opening the door to a new era of precision medicine and better global health results.

Their unique properties, such as high sensitivity, specificity, and rapid response, make them a transformative technology in healthcare. With unique physical and chemical properties, quantum effects, and higher reactivity, NMs interact with biological molecules, pathogens, and other analytes with exceptional precision. Recent advancements in nanoparticle-based biosensors have created new opportunities for accelerating diagnosis. High surface area and optical characteristics of metal and metal oxide NPs have made it possible to detect a variety of health conditions and diseases including cancer, viral infection, biomarkers, and *in vivo* imaging. Metal NPs have been produced in a variety of ways, enabling the creation of innovative tools for chemical and biological sensing targets. The utilization of various metal nano-formulations, metal oxide nano-platforms, and their composites in the early identification of illnesses have been reported.^213^ Recent outbreaks of various diseases have taught us to develop innovative materials, methods, systems and technologies for an early detection of illnesses and their effective management to minimize the losses as experienced during COVID-19 pandemic.^214^ Diagnostic strategies, thus form an important part because they can make it possible to prevent the widespread prevalence of diseases, high mortality, and recurrence after the treatment.^215^ An early diagnosis helps develop preparedness for developing control measures.^216^ Modern molecular biological methods using gene sequencing have added advantages in precise diagnosis and personalized treatment strategy.^217–219^ Advancement of NPs with respect to their sizes between 1 and 100 nm is a major focus of nanotechnology since it enables the researchers to create materials with distinctive structural and molecular properties with tuning of chemical, optical, and electrical properties.^220,221^ In bio-medical sciences, they provide the user with an effective nano-platform in order to construct the gene and drug delivery, as well as the diagnostics systems.^222,223^ Further, the nanotechnology has enabled the development of novel sensors, with increased sensitivity and specificity of operating point-of-care systems.^224^ For image-based and clinical diagnostic of respiratory viruses, NMs are emerging as promising substrates because of their unique optical, electronic, magnetic, and mechanical properties. Promising NMs for viral detection include metal, silica, and polymeric NPs, QDs, and carbon nanotube. NMs have been widely used in continuous health monitoring through wearable nanosensors devices. The example includes sweat or tear analysis for cortisol, breath analysis for carbon monoxide, nitric oxide and hydrogen sulphide using nanosensors for real-time health tracking. Based on the applications and NPs used, these have been categorized in different types.

### 7.3 Enhancement of therapeutic outcomes

**Fig. 10:**
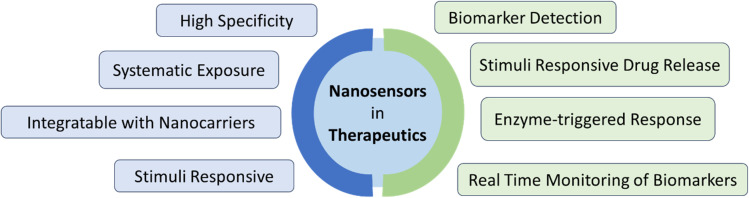
Key features and mechanism of action of nanosensors.

Nanobiotechnology is one of the most rapidly growing and promising fields. Targeted drug delivery is a rapidly evolving field that aims to deliver therapeutic agents specifically to diseased cells or tissues, minimizing the side effects and maximizing the efficacy. Modern drug delivery, monitoring drug release at targeted sites is crucial to optimize therapeutic outcomes. Such drug delivery system often utilises nanocarriers due to their numerous advantages including detection of the local environment and triggering drug release in response to specific conditions. One of the benefits of nanocarriers is the ability to manipulate their size and surface functionalization to achieve site-specific targeting. Many studies have reported their controlled and targeted drug release profiles. Nanocarriers are propelled to their target tissues, where the entrapped drugs are released, prolonging therapeutic efficacy and decreasing undesired side effects avoiding the collateral damage. Hence, these systems offer the benefits of targeted and controlled drug delivery, high bioavailability and thus improves therapeutic efficiency with simultaneously the low toxicity (shown in Fig. 10).^225^

Researchers are working on a number of different therapeutics regimens where a nanoparticle can encapsulate specific drug and helps deliver medication directly to diseased cells while minimizing the risk of damage to healthy tissues. This has the potential to change the way the clinicians treat cancer and dramatically reduce the toxic effects of chemotherapy by minimizing collateral damage. Microbial pathogens unlike antibiotics cannot develop resistance against metal particles like Ag and Au, which have anti-microbial properties targeting different components. There are research evidences for Au and Ag NPs having potential antibacterial properties against Gram negative bacteria like *E. coli* and *Mycobacterium tuberculosis*.^226^ Nanofibers are being used in wound dressings and surgical textiles, as well as in implants, tissue engineering, and artificial organ components. Scientists are working on developing ‘smart bandages’, which when left on the site, will absorb itself into the tissue once the wound heals. Embedded nanofibres in these smart bandages can contain clotting agents, antibiotics, and even sensors to detect signs of infection. Magnetic NPs have been widely used in target drug delivery system, imaging, and extraction of biomolecules, postulating them also as an important tool in cancer treatment.^227^

Nanotechnology, particularly the use of nanosensors, plays a crucial role in achieving this goal. Nanosensors being the miniature devices are capable of detecting and responding to specific biological or chemical signals, making them ideal for monitoring and controlling drug delivery systems. Nanosensors can be used in the targeted detection of various types of carcinomas such as in lungs, breast or prostate with more sensitivity and accuracy. Optical sensors can detect the difference in light emission and absorption (*e.g.*, QDs, CNTs, Au NPs), magnetic sensors detect the specific molecules, whereas the electrochemical sensors sense the changes in the electrical signals. Radiosensitization, biomarker detection, and photothermal therapy have led to targeted cancer cell destruction.^228^ Potent drugs are known to be highly toxic which can affect normal functioning cells as well. The nanobiosensors can be implemented in the routine drug monitoring at low cost for direct, rapid, and specific drug determination for drugs with low therapeutic index such as docetaxel, paclitaxel, *etc.* in body fluids like urine, blood serum. They bind to the microenvironment of receptors sites and allow targeted therapy reducing the toxicity to normal cells.^178^ For specific and targeted drug delivery, it is required to identify specific markers in cells. Identification of biomarkers like proteins and metabolites, FET based biosensors with various biomaterials like silicon nanowires, graphene and transition metal dichalcogenides to evaluate patient health and treatment efficacy in the clinical setups. They have quick responses and need no detection labels in identifying molecules.^229^ There are studies that demonstrate efficacy of ultrasensitive electrochemical aptasensor for tracking INF-γ levels in immunotherapy. It monitors the concentration before and after the treatment, indicates the response of patients to the drug therapy. Monitoring the daunorubicin *in vivo*, achieving nano-molar precision and resolution is helpful in achieving required drug delivery and dosage adjustments. The nano-probes facilitate target imaging and precise local access improving the knowledge of intracellular and extracellular environment for receptor specific treatments.^230^ Nanostructured integrate into microneedles, dendrimers, liposomes, *etc.* allow for effective real time diagnostics aiding in the delay free sensing improving accuracy and responsiveness of the therapy.^231^ Ongoing research and development efforts are paving the way for the widespread use of nanosensors in targeted drug delivery. As technology advances, we can expect to see even more sophisticated and effective nanosensor-based systems that revolutionize the way we treat diseases.

In the realm of targeted drug delivery, nanosensors offer precise control over drug release, improving therapeutic efficacy and minimizing side effects. Their ability to operate at the molecular level allows for real-time monitoring and delivery of drugs to specific sites in the body, making them a cornerstone of modern precision medicine. Key features of nanosensors in drug delivery and mechanism of action are presented in Fig. 10.

Bone and neural tissue engineering are important concern in regenerative medicine. Researchers are now looking for ways to grow complex tissues with the goal of growing human organs for transplant. Efforts are also in vogue to use graphene nanoribbons and other novel NMs to help repair spinal cord injuries. The micro-scale robots (nanobots) essentially serve as miniature surgeons can be inserted into the body to repair and replace intracellular structures.^232^ These can also replicate themselves to correct a deficiency in genetics or even eradicate diseases by replacing DNA molecules.^233^ With the help of nanobots, nanotechnology is transforming surgery by enhancing precision, minimizing invasiveness, and improving outcomes. Its applications span diagnostics, treatment, and post-surgical care, revolutionizing traditional surgical practices.^234^ Nanosensors are used for real-time monitoring during surgeries. The example includes nanosensors providing feedback on tissue oxygenation, blood pressure, pH during critical surgeries. By addressing current challenges and advancing interdisciplinary research, these technologies are poised to transform patient care and usher in a new era of personalized medicine.

Nanotechnology has become a cornerstone in modern vaccine development, offering innovative solutions to enhance vaccine efficacy, delivery, and safety.^235^ The interaction of NMs in nanoscale allows them to cross biological barrier, access new sites of to interact with DNA or proteins, a prerequisite of vaccine and drug delivery. NMs provide unique physicochemical properties, enabling precise control over antigen presentation and immune system activation. Application of nanotechnology in vaccinology emphasizes nano-carrier-based delivery with varying success.^187^ Efforts are also being made to develop multivalent vaccines for various diseases specially the respiratory viruses affecting worldwide a large population every year. One of the key contributions of nanotechnology lies in the design of antigen delivery systems, where NPs such as liposomes, polymeric carriers, and lipid NPs encapsulate antigens and ensure their efficient uptake by target immune cells. In parallel, nano-based adjuvants have been developed to enhance immune responses by stimulating specific pathways, ultimately increasing both the potency and durability of protective immunity. The most notable success of this approach is the use of lipid NP platforms in the stabilization and delivery of mRNA vaccines, exemplified by the Pfizer-BioNTech and Moderna COVID-19 vaccines.^236–238^ These lipid NP based mRNa vaccines for SARS-CoV-2 showed efficacy compared to other approaches of delivering mRNA, which is justified by their stability, efficient delivery, and intercellular transport ensured by the NPs.

**Fig. 11:**
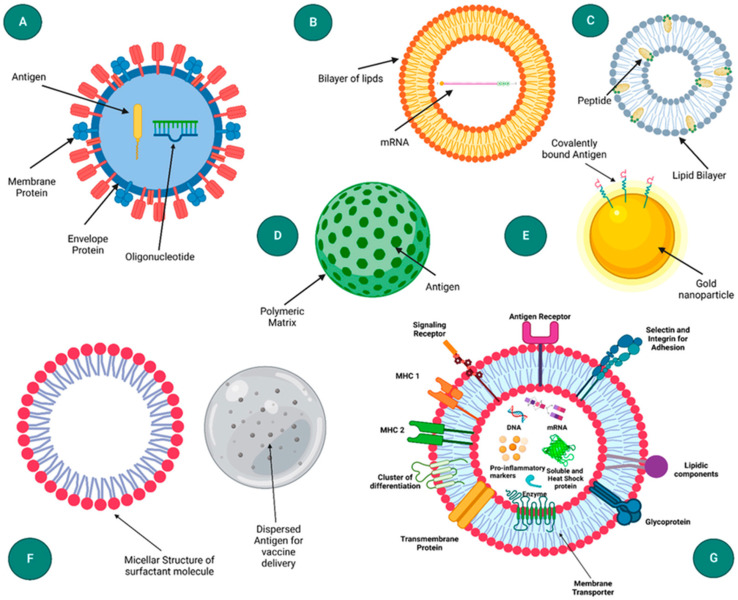
Different types of nanoparticle delivery structures in recent vaccine technologies where (A) virus-like particle, (B) liposome, (C) immune stimulating complexes, (D) polymeric nanoparticle, (E) inorganic nanoparticle, (F) emulsion and (G) exosome.^239^.

Different types of NMs that are trend in current vaccine technologies are presented schematically in Fig. 11;^239^ they include, inorganic^240^ and polymeric NPs,^241^ virus-like NPs,^242^ immune stimulating complexes.^243^ Inorganic NPs where are focused on transporting antigen expressing DNA plasmids for influenza and hepatitis B like diseases, polymeric NPs are preferred for their high biocompatibility. In contrast to the aforementioned NMs, virus-like NPs are self-assembled layer of virus capsid proteins, providing the advantage of exposing antigen on both surfaces. Colloidal saponins and lipid particles are also found to be potential in vaccine delivery systems recently. Moreover, all of these nano-carriers enable controlled and sustained antigen release, which reduces the need for frequent booster doses and improves patient compliance. Functionalization of NPs facilitates targeted delivery to specific immune cells, minimizing off-target effects and enhancing overall vaccine efficiency.

However, this efficacy is more or less influenced by the properties of NMs, *e.g.*, size, shape, and surface charge. This can be exemplified *via* CNT-based nanocarriers, whose length controls the cytokine reactions.^244^ Although NPs smaller than 200 nm can circulate through venous systems, their capacity to interact with antigen-presenting cells diminishes when they go smaller than 50 nm. Beyond delivery and targeting, nanotechnology plays a pivotal role in improving vaccine stability and storage, ensuring formulations remain effective even under challenging transportation and environmental conditions. Importantly NPs can be engineered to carry multiple antigens simultaneously, paving the way for multivalent vaccines capable of protecting against several pathogens in a single formulation—a particularly valuable approach for combating respiratory viruses that continue to affect large populations worldwide.

**Fig. 12:**
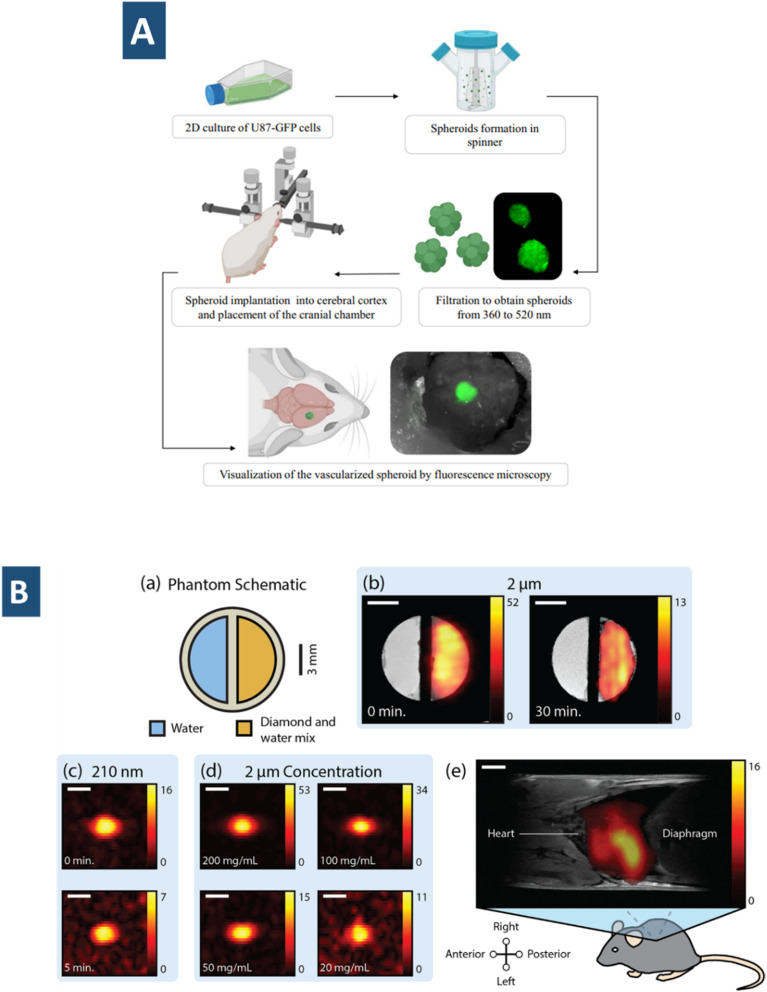
(A) Brain tumour study by AGuIX NPs grafted with a ligand peptide and integrated with porphyrin for dual mode sensing.^249^ (B) Hyperpolarized nanodiamonds for enhancement of MRI capabilities.^250^

Real-time visualization of metabolic processes and disease-related changes through non-invasive probes and detectors are the goals of bioimaging. Use of NMs as contrasting agent not only improves the accuracy of imaging, but also the size and shape dependent properties enables us to use them in different modalities ranging computed tomography (CT), magnetic resonance imaging to photoacoustic imaging.^245,246^ NMs are increasingly utilized in these technologies due to their unique physicochemical properties, offering enhanced sensitivity and specificity for biomedical applications. These are the imaging techniques with higher resolution, sensitivity, and functionality in medical, industrial, and scientific applications. By utilizing nanoscale materials and devices, it enhances imaging at the molecular and cellular levels, making it possible to visualize structures and processes with unprecedented clarity.^247,248^ A striking example of this can be found in the application of magnetic NPs. In contrast to the magnetic NPs that tend to provide high-resolution imaging, high atomic number NPs offer superior contrast for visualizing tumors by enhancing X-ray absorption in CT (Fig. 12(A and B)).^249,250^

Dual imaging modalities can be developed employing Theranostic AGuIX NPs. While functionalized with the KDKPPR peptide, NRP-1 receptors can be targeted; this act as the basis of vascular photodynamic therapy in glioblastoma treatment (Fig. 12(A)).^249^ Characterized by size of approx 11.6 nm and survival period of 13 days, they are retained in tumour vasculature for prolonged period and delay significant tumour growth. The high longitudinal relaxivity of T1-weighted MRI ensures deep tissue imaging, porphyrin photosensitizers are added to facilitate fluorescence imaging for real-time visualization of vascular retention. Long spin-lattice relaxation time in MRI provide clear imaging contrast. This has been demonstrated in the work of Waddington *et al.* who used a phase encoded hyperpolarized nanodiamonds for MRI applications. Despite the challenges of polarization decay, these nanodiamonds showed promise for targeted theranostic MRI applications (Fig. 12(B)).^250^ Thus, nanotechnology is pivotal in advancing personalized medicine, non-invasive diagnostics, and material science, bridging the gap between visualization and functional understanding.

## 8. 3D and 4D printing technologies: shaping the future of biosensors

The fourth industry revolution demands for the massive capability of biosensing devices. Such a requirement is now achievable in reality through advanced manufacturing, *i.e.*, manufacturing of biosensing elements with highly intricate and customizable features. The innovative strategy involves rapid prototyping to produce complex functional 3D or 4D objects by layering cytocompatible materials laden with living cells. The development of cost-effective, sturdy smart materials has excellent promise for usage in biomedical applications. 4D printing techniques additionally include a surrounded capability of shape transformation of 3D bioprinter structures under an external stimulus imitating the dynamic of the native tissues, organs *etc.* With three basic techniques: extrusion-based, laser-based, inkjet bioprinting. Various types of 3D and 4D printing techniques for biosensor fabrication are summarized in brief in Table 3. 3D or 4D printed objects that show shape transformation upon a favoured stimulation are of greater interest. 3D printers that employ extrusion printing use a pneumatic actuator to deliver ink through a nozzle for material deposition during object production. Prior to this, inkjet printing uses electrical actuators to expel pico-litre amounts of liquid from micron-sized nozzles onto a substrate in a predetermined pattern using a layer-by-layer process. This approach is easily adaptable to a wide range of liquid or solid solutions, including conductive polymers, dielectric inks, proteins, and living cells, and requires no post-processing. Aerosol jet printing (AJP) is another popular additive manufacturing technique for the printing of patterned circuits at microscale level with high spatial resolution. Coating of various functional NM on the substrates are being performed using AJP. Incorporation of NMs and their composites especially polymer matrix nanocomposites, ceramic matrix nanocomposites, and metal matrix nanocomposites improve the functionalities of the structures. Meanwhile, the sensing abilities along with as electrical and mechanical properties, thermal conductivity, toughness, and strength of them are enhanced.

**Table 3:** Various 3D printing technologies used in production of biosensors, analytical purposes, limits of detection and biological sample of interest

3D printing technologies	Material	Analytical purposes	Biological sample	Limit of detection	Ref.
FDM	Graphene/PLA	Detection of H_2_O_2_		11.1 μM	251
Inkjet printing	Ag NPs/SU-8	Monitoring of epithelial cell culture	Epithelial cells	4.36 cell-index unit per cells per cm^2^	252
AJP	Au NPs-PDMS	Detection of SARS-CoV-2 antigens	Human biological fluids	S1 protein: 2.8 × 10^−15^ M	253
				RBD: 16.9 × 10^−15^ M	
FDM	CNT/CB/PLA	Detection of dopamine		1.45 μg mL^−1^	253
FDM	Nanocarbon-PLA	Quantification of mucin 1	Breast cancer cells	80 nM	254
Inkjet printing-drop-on-demand printer	Graphene-PI	Fabrication of conductive ink			255
FDM	rGO-PLA	Quantification of serotonin	Synthetic biological fluids	0.032 μmol L^−1^	256
Vat polymerization	rGO-TEPA/PB	Determination of glucose	Human blood	25 μM	257
Direct-writing	Graphene nanoflakes/MWCNT/PDMS	Determination of breathing rate			258
Inkjet printing	Ce NPs-Alcohol oxidase	Measurement of alcohol level		0.001%	259
3D microfluidics	Nanoporous silicon	Detection of protein		0.04 μM	260
Inkjet printing	GO-pentacene	Detection of artificial DNA		0.1 pM	261

Detection of H_2_O_2_ became one step easier when using 3D-printed enzymatic Au NPs coated graphene–polylactic electrode.^251^ The electrode showed a LOD of 11.1 μM and a limit of quantification of 37 μM. This serves as an innovative foundation for the manufacture of third-generation electrochemical biosensors to interact without using any kind of electron mediators and binder polymers for applications in biomedical fields (*e.g.*, detect glucose and other biomarkers in biological fluids). Wang *et al.* took FDA technology to another dimension for fabricating electrochemical biosensors with the purpose of overcoming the crucial challenge of chiral recognition of molecules.^262^ The electrode of the chiral sensor has been fabricated from a Fe_3_O_4_-magnetic covalent organic framework functionalized with bovine serum albumin. The sensor showed successful recognition of l and d tryptophan isomers along with excellent repeatability for the relative quantification. By using fused deposition modelling (FDM), one of the most employed printing techniques to create complex geometries, a 3D-printed portable paper cartridge has been fabricated for quantization of drug in biological fluids.^263^ Deposition of silver nanowires at the tip of cartridge helps in optimizing the Raman signal of analytes by preconcentrating them for quantitative analysis. Consequently, their fluorescence signal, improved 9.93-fold compared to those found in multi-step existing technologies. Such printer makes it simpler and cheaper to detect broad spectrum anti-neoplastic drugs like those of epirubicin hydrochloride and cyclophosphamide. Direct ink writing, another technique for contactless printing and rapid deposition has been used by Mojena-Medina *et al.* to fabricate interdigitated-electrode sensors for monitoring cell cultures.^264^ Conductivity of PET in the printed sensor was enhanced by Ag NPs. 3D printing technologies involve multiple elements for the architecture of the device, specially the printable biological-based ink. With this notion, Parate and coworkers fabricated AJP graphene-based immunosensor which print by depositing nanoscale graphene–nitrocellulose ink.^265^ The results showed that the immunosensors were effective for monitoring cytokines in bovine serum due to their wide sensing range, low detection limit, and superior selectivity. Sensitive, rapid detection and early screening of COVID-19 is possible using 3D sensor electrodes fabricated from nanoflakes of rGO.^266^ The electrodes were integrated in a microfluidic device; the selective recognition of viral antigens was sensed from the variation in the impedance of the electrical circuit. The sensor showed LOD nearly about 16.9 × 10^−15^ M for its receptor binding domain and 2.8 × 10^−15^ M for the spike S1 protein.

Interesting developments in biosensing result from the combination of 3D printing and other methods. MIP formulation was developed by Gomez and coworkers from *N*-carbobenzyloxy-l-phenylalanine.^266^ When dansyl-l-phenylalanine is available for binding, quantitative identification is possible. An array of about 20 × 60 μm cantilever sensors were produced with these MIPs. Incubating them with analyte having fluorescent properties induced a reversible shift of the resonant frequency of the sensors; such a variation is reported to be higher than the commercial micro gravimetric equipment. Most of the cases, thermoreversible polymers are used as membrane matrices in biosensors and electroconductive hydrogels due to their favourable tensile strength and resilience. A UV-controlled bioprinting method has been developed for fabricating nanocomposite blend patch laden analogous with human coronary artery endothelial cells. The cardiac patch included CNTs incorporated with alginate hydrogel and cell-laden methacrylated collagen. Through a similar approach, 3D printed solid phase extraction sorbent has been developed for robust mycotoxin analysis, where MIP-based cylindrical poly-ε-caprolactone scaffold act as a model template to recognize ergot alkaloids.^252^

Biosensor elements sometimes require separation abilities as well as the prompt recognition of analytes. This has been possible to achieve with these printing technologies to build up systems with multi-variant actions. For instance, 3D printed microfluidic device reported by Parker and coworkers^267^ possess immunoaffinity monoliths for the extraction of preterm birth biomarkers (PTB). The glycidyl methacrylate monolith is site-specifically polymerized within the stereolithographic 3D printed device facilitating interaction of antibodies against the PTB in human serum matrix. Sorbents with scabbard-like shape has also been used for the extraction of steroids in human plasma.^268^ Colloquially, the controlled immobilization of biorecognition elements is highly expected feature of a biosensor, which is quite often a time-consuming process. A new strategy was recently exploited by Mandon *et al.* to produce functional 3D printed objects by entrapping biomolecules during the polymerization.^269^ A sandwich immunoassay has been conducted using polyethylene glycol diacrylate-based hydrogel with integrated monoclonal antibodies against the brain natriuretic peptide (BNP). Along with the peptide, the biotinylated anti-BNP antibody and alkaline phosphatase labeled streptavidin was also used during incubation; detection measures are performed by exposing the propeller to the chemiluminescent substrate that revealed the presence of BNP–anti-BNP conjugates. Using a very similar approach, photocurable formulation of bisphenol A ethoxylate diacrylate has been performed by 3D digital light processing.^270^ They can even be integrated into a monolithic lab-on-chip by stereolithographic printing process; in order to detect biotin a biotin-conjugated scaffold was used, which after integrating in a microfluidic reactor was able to perform real-time analyses.^271^

The field of wearable biosensors has seen a significant revolution because 3D printing technology, which makes it possible to fabricate complex sensor components directly onto flexible and biocompatible substrates. A flexible enzyme-electrode sensor was fabricated by means of rotated inkjet printing where Pt NPs ink was used to generate a 3D multilayered nanostructure.^272^ Nguyen *et al.* made a similar attempt in developing an amperometric biosensor to evaluate glutamate release.^273^ In this study, the nanocomposite ink was prepared from Pt NPs, MWCNTs, conductive polymers. 3D printing also has the application to fabricate electrocardiogram biosensors in order to monitor the heart impulses every time it beats. With seamless design, the sensors can be integrated into clothing or used as adhesive patches. In the work of Li *et al.* photocuring techniques has been used to develop them from 3D-printed graphene/polymer nanocomposites.^274^ Shar *et al.* developed a proof-of-concept wearable biosensor devices with 3D-printable carbon nanotube–silicone composite for advanced health monitoring and biomimetic cell culture platforms.^275^ Drop-casting method is used to fabricate the layer of 3D nanocomposite of flower α Ni(OH)_2_, Au NPs, and β rGO onto a glassy carbon electrode, which has potentiality as wearable glucose sensor with sensitivity levels of 559.314 μA mM^−1^ cm^−2^.^276^ A liver-on-a-chip glucose sensor is developed by Lee *et al.* where MWCNTs were used.^277^ Biocompatible pressure sensors from nanocomposites enables real-time health monitoring analyzing different physiological signals from respiration to artery pulse of a patient.^278^ The first ever 3D printed cerium oxide NP based colorimetric enzymatic biosensor was reported in the work of Mustafa *et al.*^259^ The breathalyzer sensor is used to determine the alcohol level in forensics.

## 9. Commercialization of nanosensors

Despite the challenges in manufacturing processes and regulatory hurdles—several nanosensor based medical devices have been commercialized so far. The growing need for cutting-edge therapeutic and diagnostic solutions is fueling the rapid growth of the developing markets for these devices. The global nanomedicine market is estimated to reach around $494 billion by 2032 with an annual growth rate of 11.3% from 2023 to 2032.^40^ Comprising over more than 200 enterprises, the drug delivery belongs 34% of the total market. Such a prevalence is thought be driven by the recent pandemic of COVID-19. Nanoparticle-based lateral flow assays have also achieved mass commercialization, most prominently in COVID-19 antigen test kits. Following is Table 4 representing a list of approved nanomedicines, assays, and kits approved by USA Food and Drug Administration (FDA), European Medicines Agency (EMA) and China's National Medical Products Administration (NMPA) in recent years.

**Table 4:** List of nanomaterial-based assays commercialized in recent years

Device name	Applicant	Year of approval	Nanomaterial	Description
Soluble ST2 protein (ST2) assay kit	Chongqing Kangjuquan Hong Biotechnology, Ltd	2024	Nanoenzyme	Fluorescent immunoassay to measure soluble suppression of tumourigenicity 2 protein (ST2) levels in human serum
Thromboelastography assay kits		2024	Nanoenzyme	Kit to detect coagulation in sodium citrate anticoagulated whole blood
Brain natriuretic peptide assay kit		2023	Nanoenzyme	Fluorescent immunoassay to measure B-type natriuretic peptide levels in human plasma
Procalcitonin/interleukin-6 (PCT/IL-6) rapid test kit	Hunan Naquan Aode Biotechnology, Ltd	2022	Nanofluorescent microsphere	Rare earth nanofluorescent kit for *in vitro* quantification of procalcitonin/interleukin 6 (PCT/IL-6) in serum, plasma, and whole blood
Cystatin C (CysC) assay kit		2022	Nanofluorescent microsphere	Nano fluorescent kit for quantifying cystatin C in human plasma
Carcinoembryonic antigen test kit		2021	Nanoenzyme	Kit for qualitative detection of carcinoembryonic antigen of breast cancer
Cardiac troponin I (cTnI) detection kit	Nanjing Botian Kezhi Biotechnology, Ltd	2021	Gold nanocages	Kit for qualitative detection of cardiac myosin-binding protein C in plasma
Urinary microalbumin detection kit	Benxi Tystj Biotechnology, Ltd	2020	CNT	Kit for qualitative urine microalbumin, used to diagnose kidney diseases

Despite these successes, large-scale commercialization faces critical hurdles: first and foremost is their scalability and reproducibility. Noble metal NPs are synthesized through expensive fabrication methods like chemical vapor deposition and lithography. Even if they have promising selectivity and sensitivity, their high susceptibility to external environment may possess threat to their performance thus impacting their targeted applications. Stability and shelf life of NMs need to be tested in stringent regulatory approval processes. Specific tests for analyzing their toxicity profiles are also essential for ensuring the safety and efficacy of these products. Biocompatibility and safety remain additional concerns, especially for devices like electronic tattoos and smart contact lenses. What is unfortunate is the cost associated with the approval processes that require significant time, posing a major challenge upon their commercialization. Multiple phase clinical trials from laboratory prototypes to large scale manufacturing require resources, manpower, and substantial investment, which the smaller enterprises may find prohibitive. Major collaborative effort between industry and academia may bridge this gap ensuring regulatory standards. Focus of research should also be on sustainability to minimize the environmental footprint of the newly produced technologies.

## 10. Navigating ethical and regulatory challenges in nanosensor development

Ethical considerations are vital to the development and application of nanosensors, while regulations offer a framework for guaranteeing their safety. The specific sets of standardized protocols and guidelines regarding the safety of patients and efficacy of the biosensors is still insufficient. The regulations for 3D nanobiosensors are inadequate as the standards to certify them is quite lengthy. The diverse nature of NMs makes it difficult to characterize, toxicity evaluation and risk assessment of the sensors according to the regulatory requirements. Biodistribution of the materials are critical factors that hinder their effectiveness and safety, which must be thoroughly understood to avoid potential toxicity due to prolonged retention. Data on potential toxicity, long-term effects, biodistribution are necessary. Most of the cases, collection of these data is time-consuming, and expensive enough to have compliance with manufacturing practices. When nanosensors pass the proof-of-concept stage, extensive testing is required across laboratories and in relevant environments to ensure their accurateness and reproducibility. The cost associated with regulatory compliance regarding preclinical and clinical studies are quite high and rarely are affordable.

There are concerns related to equity and access. As nanosensor technology advances, a considerable risk of digital divide pertains, as a consequence of which these innovations might not be equally accessible to all due to socioeconomic factors. The primary ethical concern regarding the use of nanobiosensors are related to privacy. Wearable nanosensors, collect a range of personal data when integrated into wearable devices. The collection, storage, and usage of this data should be protected from unauthorized access. A significant translational barrier exists regarding the intellectual property, such as patent protection and infringement issues which affects the commercial viability and investments on development of nanosensor. The long-term effects of NMs on ecosystems need to be assessed so that proper disposal and recycling protocols are established. The difficulties in acceptance of their potential emerge from lacking of public trust. Mass people should be engaged and acknowledged about the technology, its potential benefits, and associated risks. This can also provide valuable insights for more responsible decision-making processes. Given this scenario, international collaboration can be vital to establish harmonized standards in the evaluation and approval processes addressing existing regulatory and ethical challenges.

## 11. Challenges and limitations in nanosensor technology

Recent considerable effort of researchers in developing nanobiosensors for various healthcare application suggests that there is still opportunity to enhance the performance of biosensors through nanotechnology in terms of their specificity, selectivity, and lower detection limit. The gradual growth of world population has direct influence on the healthcare domain. Especially, healthcare is being frequently challenged with lacking of diagnosis machines, testing facilities when any pandemic surges. The progress on their development will be hindered unless they are made accessible to diverse population. However, significant challenges still pertain in the commercialization of nanobiosensors. Apart from the growing regulatory concerns and ethical needs, these challenges need to be addressed before making them commercially feasible.

Multimodal sensing, is the reliability of a sensor that can be enhanced by incorporating more than one mode of sensing to cross-validate the results. Increase in the size of the sensor and computational time are the major substitutes of multimodal sensing leading to increased cost and power consumption. Nanotechnology, in general, is a relatively novel concept, however, little experimental data are available about their harmful effects. This lack of information may lead to impediments in the safety regulation of nano-therapeutics and present before us unique assessment challenge. Numerous metal NPs, particularly those derived from Ag, Au, or Cd, have been shown to be harmful *in vitro* and *in vivo* under particular circumstances. Despite their outstanding performance *in vitro*, rigorous clinical approaches are required for their approval agencies like FDA. Thorough investigations are required considering several issues such as the stability, cellular interaction and the uptake manner in physiological conditions. There is uncertainty regarding the intricate biodistribution pathways *e.g.* circulation time, tissue accumulation, and possible long-term retention, making them difficult to anticipate. Research efforts should be more focused on improving the cytotoxic profiles of the NMs by controlling their size and surface modification as cytotoxic profiles are closely related to their size and degree of oxidation instead of looking for better suitable combination. Mediation of the interaction in physiological system through proper surface modification can be essential to ensure the biocompatibility of the materials. The standardized safety protocol should encompass safe concentration ranges and exposure limits by meticulous evaluation of their cytotoxicity and genotoxicity aspects. Control over accurate diagnosis and therapeutics can be possible with proper utilization of the endogenous and exogenous stimuli. Safety of nanosized robots should be analysed in biological systems, in accordance with the correct choice of the materials. It is high time to turn back from the *in vitro* systems to analyse the complete safety profile. Because of the popularity of personalised medicine, the concept of ‘all-in-one’ becomes applicable to the field of nanosensor too. However, this throws some of the challenges like low integrated efficiency, lack of synergistic functions, complex design, and so on. Attention should be on both their rational combination as well as the mighty purpose of multifunctionality.

Paper-based devices contain errors introduced due to the inherent variability in paper quality. Thus, high precision is hardly met in the conventional paper-based assays. The inherent qualities of paper-based microfluidic devices limit certain applications such as the cases where bead manipulation and droplet formation are required. Moreover, mechanical properties such as tensile strength and stretchability becomes a common issue, when paper-based assays are involved in stressful physical conditions. Development of assays are crucial in this regard that can be adaptable to various environments. It is also challenging to restructure the current manufacturing methods scalable enough to balance up both the cost-effectiveness and quality. Developing scalable manufacturing methods that maintain the accuracy and effectiveness of the sensors is essential. Very often, the accuracy of the tests is affected by non-uniform distribution of analytes. LFA platforms should be designed to be portable along with electronic reader instruments. Developments of new labels are necessary that are stable and efficient at the same time to ensure prompt diagnosis of infectious diseases in environments where resources are limited. Where the nano-healthcare technology is growing at leap and bounds, the diagnostic costs also surge in developing and underdeveloped countries. Development of technologies for portable POCT devices is also highly necessary. One of the main struggles to combine 3D printing with micro and nanosensor technologies is the limited range of materials that may be compatible with both procedures. The applicability of biosensors is being advanced from functional NMs like graphene, CNTs, however, 3D printing systems can hardly meet the specific functionality for these materials. Many modern 3D printers even fall short of the required level of accuracy, which causes issues with the reliability and efficiency of the sensors. Installation of nanosensors in instruments presents a number of difficulties, more commonly the packaging and miniaturization. Designing electronic circuits that require less power, and effective calibration and maintenance while reducing noise and signal interference are often challenging. Cost should be optimized by installing standalone readout devices at hospitals. Also, mass manufacturing of sensing devices and readout systems using 3D printing and machine moulding can be helpful.

There is no doubt that integration of *in vivo* nano-communication in healthcare will expand a myriad of potential services. Challenges are still remaining in developing robust largescale networks. Especially the concern in the propagation of signals; in addition, the traditional models are not supported by well-established databases that contain information of various health conditions. New coding schemes need to be developed which have compliance with the nanomachines.

## 12. The next frontier for nanosensors in healthcare

**Fig. 13:**
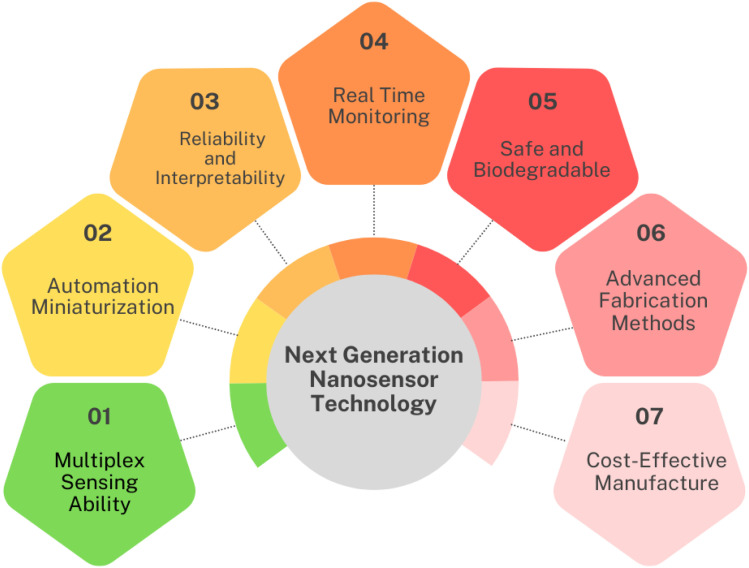
Prospects of nanosensors of next generation healthcare.

NM-based biosensors have shown improved sensitivity and selectivity, as well as faster and lower detection limits, in contemporary healthcare. However, in order to widen up the scopes for their application, not only the materials should be intelligently functionalized, but also to be chosen at right concentration along with the optimization of the performance of the sensors. With a trajectory characterized by automation, integration, and miniaturization, nanobiosensors have limitless possibilities for the future (Fig. 13). The recent pandemic teaches us to develop highly efficient and smarter healthcare technology that facilitates early diagnosis. NM based biosensors that are safe, prompt and are of low-cost can drastically lessen the dependence on the RT-PCR like expensive methods for mass testing. The sensitivity and selectivity of the biosensing platform are more or less effected by the characteristics of the biomolecules. A sensor can achieve good selectivity on optimizing the probe concentration against the bio-agent to be detected. For instance, to manage this pandemic nanobiosensors employing biomolecules like nucleic acid, protein failed to provide result with 100% accuracy due to their high sensitivity. Deep theoretical insight and rigorous research efforts are required to bring out the advancement in the development of universal biosensors that are efficient to detect analytes in different clinical sample. The sensing capabilities of the current sensors can be improved by upgrading them into multiplexed sensing for complex matrix analysis. The notion can be found in the work of Ye *et al.* for the fabrication of a nano-surface plasmon nanosensor by functionalizing Au nanorods with Hg^2+^ and Pb^2+^ aptamers for simultaneous Hg^2+^ and Pb^2+^ detection.^279^ Likewise, fluorescence and FRET signals at various wavelengths can be detected by manipulating optical sensors to identify the analyte.^280^

Despite the early stage of research, substantial work is needed to deploy paper-based microfluidics in a number of complex systems, from POC to monitoring. The applicability of the device will be directly influenced by the sustainability of the materials and their fabrication cost. Instead of including new potential features, attention should be particularly on improvement of their reliability, compatibility and easy-interpretation of results. Machine learning can be a reliable tool to analyze the analytical performance from optimizing the structure of materials. In order to accelerate the commercialisation of paper-based products, laboratories with expertise in papermaking, converting, and printing technologies may eventually play a bigger part in customising innovative, functionalised, and bioactive paper substrates as well as mass fabrication techniques. Further research in paper-based microfluidics may include developing prototypes of 3D devices that operates on capillary flow, the development of methods for mixing fluids and their integration with other microfluidic platforms. Nevertheless, the recent progress showed development of highly sensitive assays with femtomolar LOD. Paper substrates can be enabled with data analysis capabilities by incorporating microcontrollers and wireless systems. This certainly holds promise for improving the capabilities of paper-based substrates, such as real-time data transfer and remote monitoring. Catalytic property and stability enhancement, miniaturization of electronic components in LFA device should be given more attention to increase its popularity. Personalized and more predictive solutions can be obtained by ensuring their smooth interfacing with other technologies with the help of artificial intelligence and machine learning.

Specialized ink formulation, material alignment, print parameter optimization are few of the parameters need to take care of when developing 3D-devices. In near future, fabrication of multi-layer cell-laden structures will be possible from advanced printing techniques to overcome issues like tissue heterogeneity. As the health and environmental sectors move towards more environmentally friendly and sustainable sensor applications, the next generation of sensors is made to be efficient and use renewable resources. The synthesis of NPs with antimicrobial and reducing properties can be harvested by introducing different natural extracts as the precursors. A substantial genre is developing in which biological molecules with intrinsic constraints, such as issues with mass production and sensitivity to denaturation, will be replaced with synthetic NMs. Nanozymes can be dependable to address the challenges of enzyme-based sensors due to their potential catalytic properties.

## 13. Conclusion

In this era of advancements, the greater need to solve variety of problems, difficulties in health care are more evident. Smart biosensors, the greatest technological advances of time, have the potential to completely change the future landscape of diagnostics, making the process of disease management to clinical diagnosis easier. A thorough discussion of nanotechnology empowered biosensor technology has been made. Emphasis has been given explicitly on metal and metal oxides and carbon-based NMs. Their potential application in POC testing, paper-based kits and 3D devices were also highlighted. Addressing the biocompatibility and safety concerns, thorough toxicity evaluation and studies are still required to remove the current bottlenecks. Continuous research effort on nano-empowered biosensor is likely to occur a significant transformation where therapies are not only efficient but also customized to particular needs of each patient. This review may be helpful to the researchers to understand the contribution of nanotechnology and finding out the gaps in existing healthcare.

## Conflicts of interest

There are no competing interests to influence the work reported in this paper.

## Data Availability

No primary research results, software or code have been included and no new data were generated or analysed as part of this review.
